# The PFC-LH-VTA pathway contributes to social deficits in IRSp53-mutant mice

**DOI:** 10.1038/s41380-023-02257-y

**Published:** 2023-09-20

**Authors:** Young Woo Noh, Yangsik Kim, Soowon Lee, Yeonghyeon Kim, Jae Jin Shin, Hyojin Kang, Il Hwan Kim, Eunjoon Kim

**Affiliations:** 1 Department of Biological Sciences, Korea Advanced Institute of Science and Technology (KAIST), Daejeon, 34141 Korea; 2 Department of Psychiatry, Inha University Hospital, Incheon, 22332 Korea; 3 Graduate School of Medical Science and Engineering, KAIST, Daejeon, 34141 Korea; 4 Center for Synaptic Brain Dysfunctions, Institute for Basic Science, Daejeon, 34141 Korea; 5 Division of National Supercomputing, Korea Institute of Science and Technology Information (KISTI), Daejeon, 34141 Korea; 6 Department of Anatomy and Neurobiology, University of Tennessee Health Science Center, Memphis, TN 38163 USA

**Keywords:** Neuroscience, Physiology

## Abstract

Dopamine (DA) neurons in the ventral tegmental area (VTA) promote social brain functions by releasing DA onto nucleus accumbens neurons, but it remains unclear how VTA neurons communicate with cortical neurons. Here, we report that the medial prefrontal cortex (mPFC)-lateral hypothalamus (LH)-VTA pathway contributes to social deficits in mice with IRSp53 deletion restricted to cortical excitatory neurons (*Emx1-Cre;Irsp53*^*fl/fl*^ mice). LH-projecting mutant mPFC neurons display abnormally increased excitability involving decreased potassium channel gene expression, leading to excessive excitatory synaptic input to LH-GABA neurons. A circuit-specific IRSp53 deletion in LH-projecting mPFC neurons also increases neuronal excitability and induces social deficits. LH-GABA neurons with excessive mPFC excitatory synaptic input show a compensatory decrease in excitability, weakening the inhibitory LH^GABA^-VTA^GABA^ pathway and subsequently over-activating VTA-GABA neurons and over-inhibiting VTA-DA neurons. Accordingly, optogenetic activation of the LH^GABA^-VTA^GABA^ pathway improves social deficits in *Emx1-Cre;Irsp53*^*fl/fl*^ mice. Therefore, the mPFC-LH^GABA^-VTA^GABA^-VTA^DA^ pathway contributes to the social deficits in *Emx1-Cre;Irsp53*^*fl/fl*^ mice.

## Introduction

Many neuropsychiatric conditions, including schizophrenia, autism spectrum disorder (ASD), and attention-deficit/hyperactivity disorder (ADHD), are linked to social deficits. Neural circuits underlying brain disease-related social deficits have been explored in multiple mouse models, and such studies have identified social circuits involving key brain regions, including the medial prefrontal cortex (mPFC), ventral tegmental area (VTA), nucleus accumbens, and amygdala [1–7]. For example, optogenetic activation of parvalbumin neurons in the mPFC normalizes pyramidal neuronal excitability and social deficits in *Cntnap2*-mutant mice [1], which is consistent with the finding that an increased excitation/inhibition ratio in the mPFC can cause social deficits in wild-type (WT) mice [8]. Optogenetic activation of VTA dopaminergic neurons, which regulates nucleus accumbens neurons [9], normalizes social deficits in mice lacking Shank3 [2], which is an excitatory postsynaptic scaffold protein implicated in ASD [10–13]. In addition, dysfunction of VTA dopaminergic neurons causes social deficits in neuroligin-3-mutant mice [3]. The PFC-amygdala pathway, which is known to regulate social functions [14], has also been linked to social deficits in Pten-, Shank3-, and ArpC3-mutant mice [4, 15, 16]. More recently, the mPFC-VTA pathway has been shown to underlie social deficits in socially isolated female mice [17]. However, it is not fully understood how abnormal interactions between PFC and subcortical areas impair social cognition and behavior in animal models with social deficits.

Despite the above findings, it has proven difficult to gain a thorough understanding of defective social circuits in mouse models of brain diseases, partly because the causal genes are typically expressed widely across brain regions and the primary deficits in a brain region often induce secondary changes in connected remote brain regions. A potential strategy for circumventing these difficulties would be to restrict gene targeting to specific brain regions and cell types, and then determine whether these restricted gene deletions lead to some—or all—of the phenotypes induced by global gene knockouts (KOs). This approach has allowed the more precise identification of brain areas and cell types associated with social deficits [18–21], but further work is still needed.

IRSp53 (encoded by *BAIAP2*) is an abundant adapter/scaffolding protein at excitatory postsynaptic sites; it regulates synaptic structure and function through Rac/Cdc42 small GTPases, which modulate actin filaments [13, 22] and thereby act on the main cytoskeleton of dendritic spines [23]. IRSp53 also regulates synapse assembly and function by interacting with other abundant excitatory postsynaptic proteins, such as PSD-95 and Shank, that have been implicated in various brain disorders, including ASD [10–12, 24, 25]. Human mutations in IRSp53 have been associated with multiple brain diseases, including ASD [26–28], schizophrenia [29, 30], and ADHD [31, 32]. IRSp53-mutant mice show social and cognitive deficits [33–36] through various mechanisms, including abnormally increased N-methyl-D-aspartate receptor (NMDAR) function [33, 37–39]. A conditional IRSp53 deletion restricted to cortical excitatory neurons (*Emx1-Cre;Irsp53*^*fl/fl*^) yields social deficits accompanying decreased excitatory synaptic transmission, NMDAR hyperfunction, and increased neuronal excitability in layer 5 pyramidal neurons of mPFC [35], which have been implicated in social and cognitive modulations [40]. Despite these findings, however, it remains unclear how this decreased excitatory synaptic function leads to increased neuronal excitability and, more importantly, whether and how the increased excitability of the mutant mPFC neurons disrupts downstream neural pathways to induce social and cognitive deficits.

In the present study, we explored the mPFC-LH^GABA^-VTA^GABA^-VTA^DA^ pathway to better understand social deficits in *Emx1-Cre;Irsp53*^*fl/fl*^ mice. We found frequent abnormal synaptic/neuronal changes in this pathway and causally associated social deficits by experimental approaches, including circuit-specific gene deletion and optogenetic and chemogenetic modulations.

## Methods

### Animals

Mice were bred and maintained according to the Animal Research Requirements of KAIST, and all procedures were approved and followed by the Committee of Animal Research at KAIST (KA2022-059). All mice were fed ad libitum, and caged under a 12-h light/dark cycle. Mice were weaned at postnatal day 21, and two to six littermates were housed together, without genotype separation, until experiments. We used male mice for behavioral, electrophysiological, and other experiments including RNA sequencing and biochemical histology. Mice of C57BL/6J background carrying floxed exons 4–6 of the *BAIAP2* gene (termed *Irsp53*^*fl/fl*^*)* were generated commercially by Biocytogen, as described previously [35].

### Behavioral assays

All behavioral assays were performed using age-matched C57BL/6J male mice (8–16 weeks) generated by Cre/+;*Irsp53*^*fl/fl*^ x *Irsp53*^*fl/fl*^ mating. Mice were handled by experienced experimenters for three days (10 min/day) to reduce anxiety-like responses. Mice were allowed to rest at least one day between tests. Behavioral tests were conducted during the light-off period. Mouse behaviors were recorded as video files and analyzed with EthoVision XT 13 (Noldus, Netherlands) (see *Supplementary Information* for further details).

### Electrophysiology

To prepare mPFC tissue sections for electrophysiological experiments, we used N-Methyl-D-glucamine (NMDG)-based artificial cerebrospinal fluid (ACSF) as a section buffer. To prepare tissue sections for LH and VTA regions, we used sucrose-based ACSF (see *Supplementary Information* for further details of tissue slice preparation). Miniature excitatory postsynaptic currents (mEPSCs) were recorded in the presence of AP5 (50 μM) and tetrodotoxin (1 μM) at the holding potential of −70 mV. Miniature inhibitory postsynaptic currents (mIPSCs) were recorded at 0 mV. To measure action potential (AP) thresholds, a series of current steps (2 ms duration at 2.5 Hz, 0–2500 pA, +10 pA step increments) were injected into patched neurons until an AP was generated. To measure input resistances, hyperpolarizing current steps (1 s duration, 0 to −75 pA, −25 pA step increments) were injected into patched neurons. All voltage measures were taken after neurons had reached a stable response. Optogenetically evoked EPSCs and IPSCs (oEPSCs and oIPSCs) were recorded at the holding potential of −70 mV and 0 mV, respectively (see *Supplementary Information* for further details).

### Stereotaxic brain surgery and viral vectors

In brief, craniotomy was made bilaterally above the mouse brain region of interest using a dental drill, using the following coordinates; LH (from Bregma, anteroposterior (AP) − 1.3 mm, lateral ±1.0 mm, dorsoventral −5.0 mm) [41]. AAV5 virus solution was infused at a rate of 0.1 μl per min (see *Supplementary Information* for further details).

### Statistical analysis

Statistical data analysis was performed using Prism 6 (GraphPad). Data normality was determined using the Shapiro-Wilk normality test. Data with normal distribution were analyzed using the Student’s *t* test and analysis of variance (ANOVA) with post-hoc tests. Data failing the normality test were analyzed using the Mann–Whitney test. The ROUT method was applied to exclude outliers, using a Q coefficient of 1%. Exact numbers of mice used and statistical details are presented in Source Data 1.

## Results

### VTA and LH regions receive inputs from multiple cortical areas

**Fig. 1:**
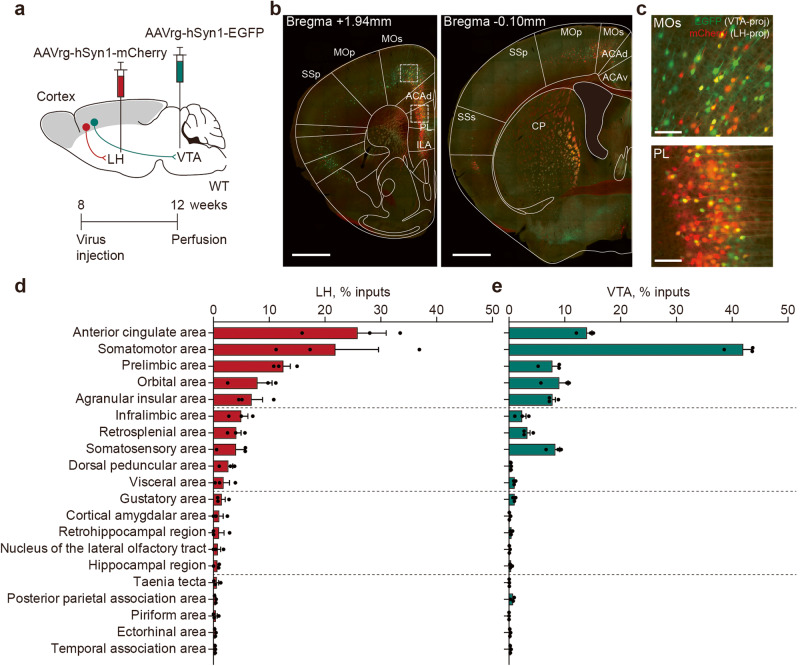
The VTA and LH receive inputs from multiple cortical areas. **a** A strategy to identify cortical regions that project to the lateral hypothalamus (LH) and ventral tegmental area (VTA) regions by performing retrograde labeling with AAVrg-hSyn1-mCherry and AAVrg-hSyn1-EGFP, respectively. Gray areas represent cortical regions where mCherry and EGFP signals were analyzed. **b**, **c** Examples of LH- and VTA-projecting cortical neurons retrogradely labeled by mCherry and EGFP, respectively. The occasional yellow-labeled neurons likely represent VTA-projecting cortical neurons that form en passant synapses in the LH. Images in (**c**) are from the insets in (**b**). SSp primary somatosensory area, SSs supplemental somatosensory area, MOp primary somatomotor area, MOs secondary somatomotor area, ACAd anterior cingulate area dorsal part, ACAv anterior cingulate area ventral part, CP caudoputamen, PL prelimbic area, ILA infralimbic area. Scale bars, 1 mm (**b**) and 100 µm (**c**). **d** Quantitative analysis of LH-projecting cortical neurons. (*n* = 3 mice). **e** Quantitative analysis of VTA-projecting cortical neurons. (*n* = 3 mice). Error bars represent the standard errors of means (sem).

In order to identify brain regions projecting to VTA neurons, we set out to perform retrograde labeling of VTA-projecting neurons by injecting AAVrg-hSyn1-EGFP to the VTA of WT mice (8–12 weeks) (Fig. 1a–c). We further sought to identify LH-projecting brain regions using AAVrg-hSyn1-mCherry, because LH-GABA neurons project to and inhibit VTA-GABA neurons [42–44].

LH- and VTA-projecting neurons retrogradely labeled by mCherry and EGFP, respectively, were analyzed using the AMaSiNe program [45]. The signals were detected in various cortical areas, including mPFC regions such as the anterior cingulate area (ACA), somatomotor area (MO), prelimbic area (PL), orbital area, and infralimbic area (Fig. 1d, e). Occasionally, VTA-projecting mPFC neurons that formed en passant synapses in the LH could be observed, as previously described [46]. These results suggest that the LH and VTA receive inputs from multiple and overlapping cortical areas, including the mPFC.

### *Emx1-Cre;Irsp53*^*fl/fl*^ prelimbic layer 5 pyramidal neurons show increased neuronal excitability

VTA and LH regions receive strong inputs from mPFC sub-regions, including the ACA, MO, and PL (Fig. 1). In addition, layer 5 pyramidal neurons in the *Emx1-Cre;Irsp53*^*fl/fl*^ prelimbic area show increased excitability [35]. We thus tested if the excitability of pyramidal neurons was altered in other cortical regions of *Emx1-Cre;Irsp53*^*fl/fl*^ mice, namely prelimbic layer 2/3 and ACA and MOs (secondary MO) layers 2/3 and 5.

Prelimbic layer 2/3 pyramidal neurons in *Emx1-Cre;Irsp53*^*fl/fl*^ mice showed normal excitability, as supported by analyses of current-firing curves, AP thresholds, and input resistances (Supplementary Fig. 1a–c). This contrasted with the increased excitability previously reported for mutant layer 5 neurons [35].

ACA layers 2/3 and 5 neurons in the mutant mice showed normal excitability (Supplementary Fig. 1d–i). MOs layer 2/3 neurons also showed normal excitability, whereas MOs layer 5 neurons showed moderately decreased excitability (Supplementary Fig. 1j–o).

These results suggest that IRSp53 deletion leads to cortical region- and layer-differential changes in neuronal excitability; increased excitability is observed in prelimbic layer 5 pyramidal neurons but not prelimbic layer 2/3 pyramidal neurons, while the ACA and MOs show largely normal excitability in layers 2/3 and 5, with the exception of MOs layer 5 neurons.

### LH-projecting *Emx1-Cre;Irsp53*^*fl/fl*^ prelimbic neurons show increased excitability

The increased excitability of layer 5 prelimbic neurons in *Emx1-Cre;Irsp53*^*fl/fl*^ mice suggest that their output to target brain regions, such as the LH and VTA, is increased. We focused on the mPFC-LH pathway because it regulates social brain functions in a sexually dimorphic manner [5] and LH-GABA neurons regulate VTA-GABA neurons under social and reward contexts [42–44].

**Fig. 2:**
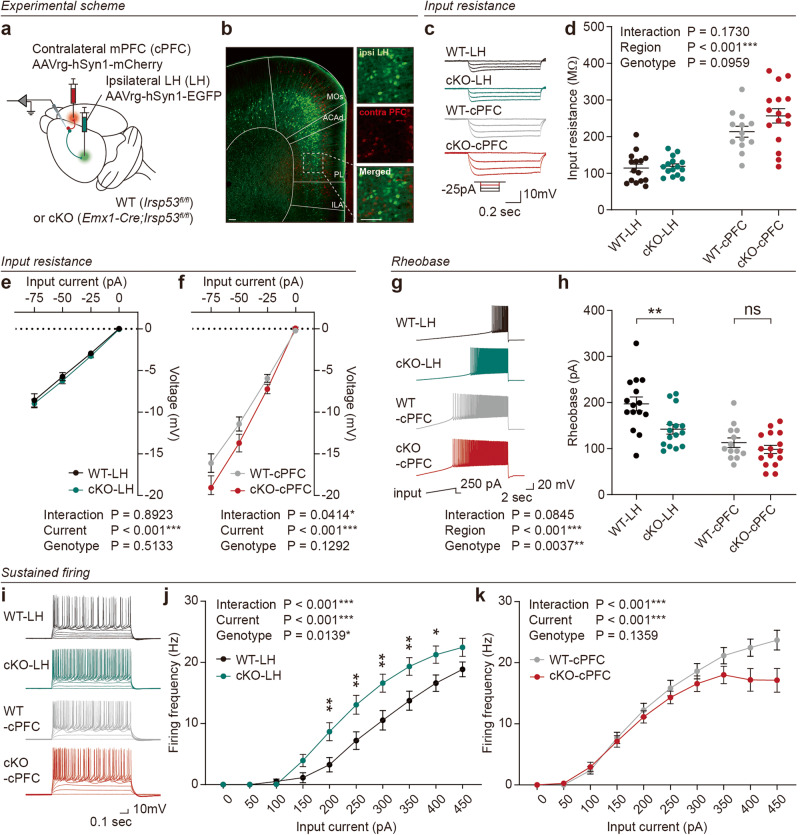
LH-projecting *Emx1-Cre;Irsp53*^*fl/fl*^ prelimbic neurons show increased excitability. **a** A strategy for retrograde labeling of LH- and contralateral PFC (cPFC)-projecting prelimbic neurons by injection of AAVrg-hSyn1-EGFP and AAVrg-hSyn1-mCherry into the LH and cPFC regions (8–12 weeks), respectively, of *Emx1-Cre;Irsp53*^*fl/fl*^ (cKO) mice. *Irsp53*^*fl/fl*^ mPFC mice were used as WT controls. **b** Examples of retrogradely labeled LH- and cPFC-projecting prelimbic neurons visualized by EGFP and mCherry, respectively. Scale bar, 200 µm. **c**–**f** Input resistance of LH- and cPFC-projecting prelimbic layer 5 pyramidal neurons in WT and cKO mice. (*n* = 15 neurons from 3 mice [WT-LH], 15, 3 [cKO-LH], 13, 3 [WT-cPFC], 16, 3 [cKO-cPFC], two-way ANOVA). **g**, **h** Rheobase results of LH- and cPFC-projecting prelimbic layer 5 pyramidal neurons in WT and cKO mice (*n* = 15, 3 [WT-LH], 15, 3 [cKO-LH], 13, 3 [WT-cPFC], 16, 3 [cKO-cPFC], two-way ANOVA with Sidak’s multiple comparison test). **i**–**k** Current-firing curves of LH- and cPFC-projecting prelimbic layer 5 pyramidal neurons in WT and cKO mice. (*n* = 15, 3 [WT-LH], 15, 3 [cKO-LH], 13, 3 [WT-cPFC], 16, 3 [cKO-cPFC], two-way ANOVA). Significance is indicated as * (<0.05), ** (<0.01), *** (<0.001), or ns (not significant).

To determine the excitability of LH-projecting prelimbic layer 5 pyramidal neurons in *Emx1-Cre;Irsp53*^*fl/fl*^ mice, we used mPFC neurons that were retrogradely labeled by injection of AAVrg-hSyn1-EGFP to the LH (Fig. 2a, b). For comparison, we characterized prelimbic layer 5 neurons projecting to the contralateral PFC (cPFC) using AAVrg-hSyn1-mCherry. LH-projecting prelimbic neurons showed increased excitability, as supported by analysis of the current-firing curve and rheobase, but not input resistance (Fig. 2c–j). In contrast, cPFC-projecting prelimbic neurons showed normal excitability. This suggested the presence of circuit-specific neuronal excitability. There was no genotype difference in any other measure of neuronal excitability, such as AP-related parameters, among LH- and cPFC-projecting *Emx1-Cre;Irsp53*^*fl/fl*^ prelimbic neurons (Supplementary Fig. 2).

These results collectively suggest that LH-projecting, but not cPFC-projecting, mPFC prelimbic layer 5 pyramidal neurons in *Emx1-Cre;Irsp53*^*fl/fl*^ mice show increased excitability in a circuit-specific manner.

### Transcriptomic changes involving potassium channel genes in the *Emx1-Cre;Irsp53*^*fl/fl*^ mPFC

We speculated that the increased excitability of LH-projecting *Emx1-Cre;Irsp53*^*fl/fl*^ mPFC neurons might involve compensatory transcriptional changes induced by the loss of IRSp53 [35], which is a key excitatory postsynaptic scaffolding/adapter protein [22]. To better understand the mechanic basis of the neuronal hyperexcitability, we attempted RNA-Seq-based transcriptomic analysis of the mutant mPFC.

The analysis of differentially expressed genes (DEGs) between mutant/cKO and WT mPFCs yielded relatively few DEGs, making it difficult to extract related biological functions (Supplementary Fig. 3a; Supplementary Tables 1 and 2). We thus attempted gene set enrichment analysis (GSEA), which uses the total list of ranked transcripts to enable unbiased identification of biological functions [47].

The results indicated that there was a strong negative enrichment (or downregulation) of cKO/WT transcripts for molecular functions associated with channels and ion transporters, as shown by the top-five enriched gene sets and clusters of enriched gene sets derived from Cytoscape App EnrichmentMap analyses (Supplementary Fig. 4a, b; Supplementary Table 3). In contrast, the positive enrichments for molecular functions were relatively weak, particularly in the clustered gene sets. GSEA for cellular components and biological processes revealed moderate enrichments for ribosome- and peptidase-related gene sets that seem relatively unlikely to be related to neuronal excitability (Supplementary Fig. 3b–e).

A leading-edge analysis was performed to identify key genes shared by the enriched gene sets [47]. This analysis returned a number of potassium channel genes found in the down-regulated cluster, but not in the up-regulated cluster, of channel/transporter-related gene sets (molecular function) (Supplementary Fig. 4c, d). These leading-edge genes included KCNJ10, KCNC1, KCNK3, KCNA2, and KCNK5. A quantitative PCR analysis for KCNK3 and KCNK5, known to regulate neuronal excitability [48, 49], revealed decreases in the expression of these genes in the mPFC (Supplementary Fig. 5a). In contrast, KCNQ2 and KCNQ3, also known to regulate neuronal excitability [50–53], were not altered in their mRNA levels. Notably, the MO region displayed differential changes in the mRNA levels of these genes while expressing comparable levels of IRSp53 mRNAs, as compared with the mPFC (Supplementary Fig. 5b, c), likely explaining the differential changes in the excitability of mPFC, ACA, and MOs neurons (Supplementary Fig. 1). Contrary to the abovementioned leading-edge analysis results based on molecular function, leading-edge analysis based on cellular component or biological pathway did not yield genes directly associated with neuronal excitability (Supplementary Fig. 6).

These results collectively suggest that IRSp53 deletion restricted to cortical excitatory neurons induces transcriptomic changes related to various biological functions, including potassium channel gene downregulations.

### Circuit-selective IRSp53 deletion in LH-projecting mPFC neurons increases excitability and induces social deficits

**Fig. 3:**
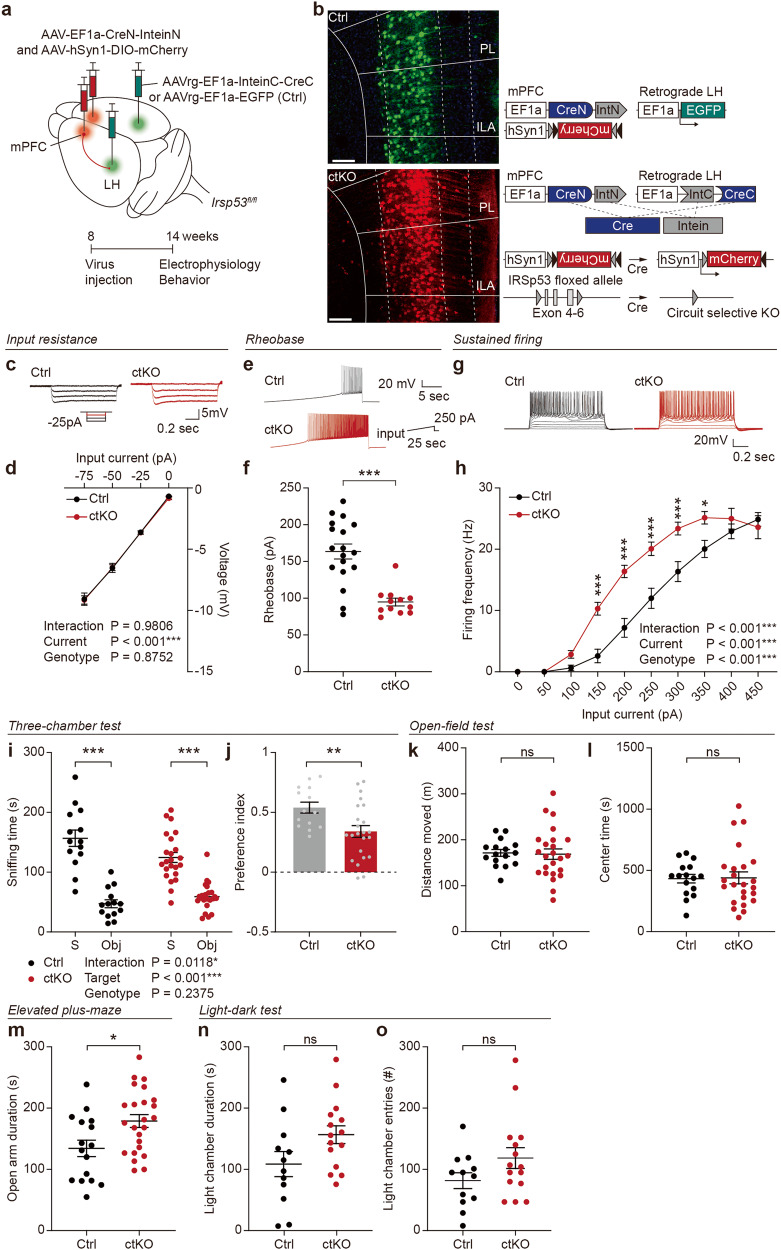
Circuit-selective IRSp53 deletion in LH-projecting prelimbic neurons increases excitability and induces social deficits. **a** Strategy for circuit-selective IRSp53 deletion in LH-projecting mPFC neurons by injection of AAV(PHP.eB)-EF1a-CreN-InteinN and AAV5-hSyn1-DIO-mCherry in the mPFC and AAVrg-EF1a-InteinC-CreC (or AAVrg-EF1a-EGFP as control) in the LH of *Irsp53*^*fl/fl*^ mice (8–14 weeks). **b** Details of the viral constructs used for circuit-selective IRSp53 deletion. Note that intein reconstitution leads to Cre reconstitution. **c**, **d** Normal input resistance in prelimbic LH-projecting layer 5 pyramidal neurons in IRSp53-ctKO mice with circuit-selective IRSp53 KO in LH-projecting neurons. (*n* = 20 neurons from 3 mice [control], 13, 3 [ctKO], two-way ANOVA). **e**, **f** Decreased rheobase in prelimbic LH-projecting layer 5 pyramidal neurons of IRSp53-ctKO mice. (*n* = 18, 3 [control], 12, 3 [ctKO], Student’s *t* test). **g**, **h** Increased current-firing curve in prelimbic LH-projecting layer 5 pyramidal neurons of IRSp53-ctKO mice. (*n* = 17, 3 [control], 13, 3 [ctKO], two-way ANOVA). **i**, **j** Moderately decreased social approach of IRSp53-ctKO mice in the three-chamber test, as supported by the preference index (S1–O over S1 + O) and time spent sniffing social/object targets (S, social stranger; Obj, object); a positive genotype x target interaction is obtained by two-way ANOVA, partly supporting the difference between WT and mutant mice. (*n* = 14 mice [control], 23 [ctKO], two-way ANOVA with Sidak’s multiple comparison test [S/O sniffing], Student’s *t* test [preference index]). **k** Normal locomotor activity of IRSp53-ctKO mice in the open-field test, as shown by total distance moved. (*n* = 16 [control], 24 [ctKO], Student’s *t* test). **l** Normal anxiety-like behavior of IRSp53-ctKO mice in the open-field test, as shown by time spent in the center region. (*n* = 16 [control], 24 [ctKO], Student’s *t* test). **m** Anxiolytic-like behavior of IRSp53-ctKO mice in the elevated plus-maze test, as shown by time in open arms. (*n* = 16 [control], 24 [ctKO], Student’s *t* test). **n**, **o** Normal anxiety-like behavior of IRSp53-ctKO mice in the light-dark test, as shown by time spent in and entries to the light chamber. (*n* = 12 [control], 15 [ctKO], Student’s *t* test). Significance is indicated as * (<0.05), ** (<0.01), *** (<0.001), or ns (not significant).

Our results indicate that LH-projecting mutant mPFC neurons display increased excitability in a pathway-specific manner. To test if this is caused by the IRSp53 deletion in LH-projecting mPFC neurons in a cell-autonomous manner, we attempted circuit-selective IRSp53 deletion in mPFC neurons using the split-intein-mediated split-Cre system (termed ctKO, for circuit-selective KO) [16]. To this end, we injected the mPFC of *Irsp53*^*fl/fl*^ mice with AAV-EF1a-CreN-InteinN and AAV5-hSyn1-DIO-mCherry and the LH region with AAVrg-EF1a-InteinC-CreC (or AAVrg-EF1a-EGFP as control) (Fig. 3a, b). This led to Cre reconstitution-dependent IRSp53 deletion and mCherry expression in LH-projecting mPFC neurons, but no Cre recombination in control neurons expressing EGFP.

IRSp53-ctKO LH-projecting layer 5 pyramidal neurons in the prelimbic area displayed increased excitability, as supported by rheobase and current-firing curve results, although not by input resistance (Fig. 3c–h). This was similar to the increased excitability of LH-projecting prelimbic layer 5 pyramidal neurons seen in *Emx1-Cre;Irsp53*^*fl/fl*^ mice (Fig. 2).

IRSp53-ctKO mice showed moderately impaired social approach in the three-chamber test, as supported by the social preference index and time spent sniffing social/object targets (genotype x target interaction) (Fig. 3i, j), suggesting the presence of partial social deficits. Open-field locomotion was unaltered in these mice, but moderate anxiolytic-like behavior was observed; significant changes were found in the elevated plus-maze test, but not the open-field and light-dark tests (Fig. 3k–o).

These results collectively suggest that circuit-selective IRSp53 deletion in LH-projecting mPFC neurons increases the excitability of prelimbic layer 5 pyramidal neurons and induces social deficits in WT mice in a cell-autonomous manner.

### *Emx1-Cre;Irsp53*^*fl/fl*^ LH-GABA neurons show increased excitatory synaptic transmission but decreased excitability

We speculated that the increased excitability of LH-projecting mPFC neurons in *Emx1-Cre;Irsp53*^*fl/fl*^ mice may increase the excitatory synaptic input to target LH neurons. To test this idea, we measured excitatory and inhibitory synaptic transmissions in LH-GABA neurons, which are known to inhibit VTA-GABA neurons for social and reward modulations [42–44].

**Fig. 4:**
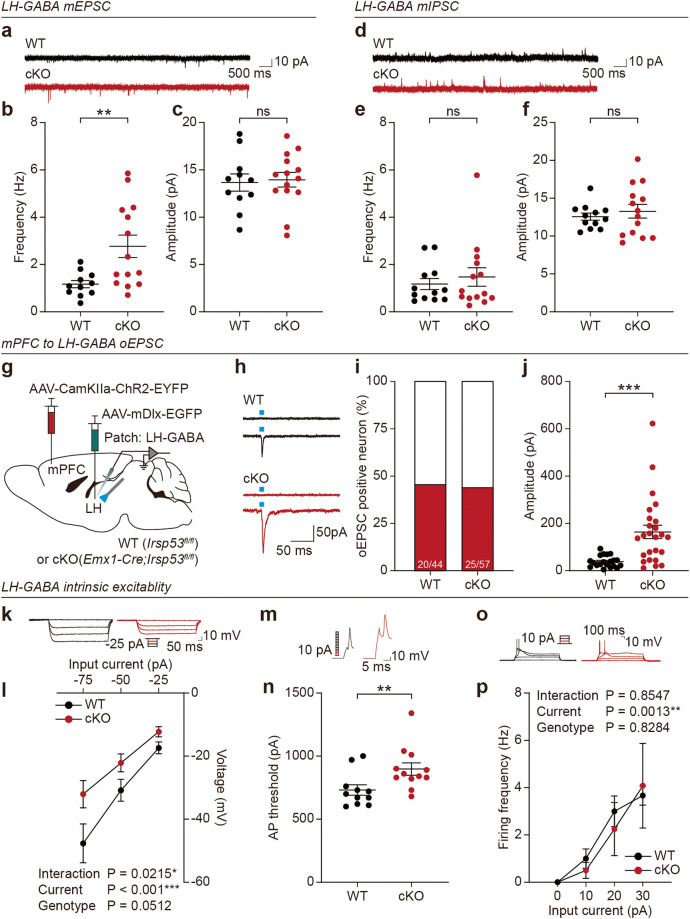
*Emx1-Cre;Irsp53*^*fl/fl*^ LH-GABA neurons show increased excitatory synaptic transmission but decreased excitability. **a**–**c** Increased mEPSC frequency but not amplitude in LH-GABA neurons in *Emx1-Cre;Irsp53*^*fl/fl*^ mice (8 weeks). (*n* = 11 neurons from 3 mice [WT], 14, 4 [cKO], Student’s *t* test). **d**–**f** Normal mIPSC frequency and amplitude in *Emx1-Cre;Irsp53*^*fl/fl*^ LH-GABA neurons (8 weeks). (*n* = 12, 3 [WT], 14, 4 [cKO], Student’s *t* test). **g** Strategy to measure optogenetically evoked excitatory postsynaptic currents (oEPSCs) in LH-GABA neurons receiving mPFC inputs. AAV-CamKIIa-hChR2-EYFP was injected into the mPFC of *Emx1-Cre;Irsp53*^*fl/fl*^ or WT (*Irsp53*^*fl/fl*^) mice, and AAV (PHP.eB)-mDlx-EGFP was injected into the LH to mark GABA neurons (8–12 weeks). **h**, **i** Examples of oEPSCs, which were observed in ~45% of light-stimulated LH-GABA neurons. (*n* = 44, 4 [WT], 57, 4 [cKO]). **j** Increased oEPSC amplitude in *Emx1-Cre;Irsp53*^*fl/fl*^ LH-GABA neurons (12 weeks). (*n* = 20, 4 [WT], 25,4 [cKO], Student’s *t* test). **k**, **l** Normal input resistance in *Emx1-Cre;Irsp53*^*fl/fl*^ LH-GABA neurons (8–12 weeks). (*n* = 11, 3 [WT], 12, 3 [cKO], two-way ANOVA with Sidak’s multiple comparison test). **m**, **n** Increased AP threshold in *Emx1-Cre;Irsp53*^*fl/fl*^ LH-GABA neurons (8–12 weeks). (*n* = 11, 3 [WT], 12, 3 [cKO], Mann–Whitney test). **o**, **p** Normal current-firing curve of *Emx1-Cre;Irsp53*^*fl/fl*^ LH-GABA neurons (8–12 weeks). (*n* = 9, 3 [WT], 12, 3 [cKO], two-way ANOVA). Significance is indicated as * (<0.05), ** (<0.01), *** (<0.001), or ns (not significant).

In *Emx1-Cre;Irsp53*^*fl/fl*^ mice, LH-GABA neurons, which can be identified by unique electrophysiological and morphological criteria [54], showed an increased frequency of mEPSCs without any change in amplitude, as compared with WT mice (Fig. 4a–c). In contrast, mIPSCs were comparable between the genotypes (Fig. 4d–f). LH-GABA neurons from female *Emx1-Cre;Irsp53*^*fl/fl*^ mice, which do not show social deficits [35], exhibited normal frequency but decreased amplitude of mEPSCs (Supplementary Fig. 7), which differed from the increased frequency (not amplitude) of mEPSCs in the male mutant mice.

To more directly test if the excitatory mPFC-LH pathway was altered in the mutant mice, we measured optogenetically induced excitatory postsynaptic currents (oEPSCs) in mutant LH-GABA neurons receiving inputs from mPFC neurons. To this end, we injected AAV-mDlx-EGFP in the LH and AAV-CamKIIa-hChR2-EYFP in the mPFC and stimulated LH-GABA neurons with blue light (Fig. 4g). oEPSCs were observed in ~45% of light-stimulated neurons; their amplitude was greater in *Emx1-Cre;Irsp53*^*fl/fl*^ LH-GABA neurons compared to control (*Irsp53*^*fl/fl*^) neurons (Fig. 4h–j).

In addition to assessing excitatory synaptic transmission, we also tested whether the excitability of mutant LH-GABA neurons was altered. Intriguingly, the excitability was decreased, as supported by the AP threshold but not by input resistance or current-firing curve (Fig. 4k–p). Similarly, circuit-selective deletion of IRSp53 in LH-projecting neurons decreased the excitability of LH-GABA neurons, as supported by the input resistance, AP threshold, and current-firing curve (Supplementary Fig. 8).

For comparison, we also characterized the electrophysiological properties of nucleus accumbens neurons in the ventral striatum of *Emx1-Cre;Irsp53*^*fl/fl*^ mice, as these neurons also receive inputs from the mPFC and other social behavior-related brain regions [9, 55, 56]. These neurons showed increased mEPSC amplitude but no change in mEPSC frequency, mIPSC frequency/amplitude, or neuronal excitability (Supplementary Fig. 9). The increased mEPSC amplitude in nucleus accumbens neurons differed from the result obtained in LH-GABA neurons (increased mEPSC frequency); this is unlikely to involve the increased output of nucleus accumbens-projecting mPFC neurons, which would increase the frequency but not the amplitude of mEPSCs.

These results collectively suggest that IRSp53 deletion restricted to cortical excitatory neurons abnormally activates the mPFC-LH^GABA^ pathway and induces a compensatory decrease in the excitability of LH-GABA neurons.

### *Emx1-Cre;Irsp53*^*fl/fl*^ VTA-GABA neurons show decreased inhibition by LH-GABA neurons

Given that LH-GABA neurons inhibit VTA-GABA neurons for social and reward modulations [42–44], we speculated that the decreased excitability of LH-GABA neurons in *Emx1-Cre;Irsp53*^*fl/fl*^ mice may insufficiently inhibit VTA-GABA neurons. To test this idea, we first measured spontaneous synaptic transmissions in the mutant VTA-GABA neurons, which can be identified by their unique electrophysiological and morphological properties [2].

**Fig. 5:**
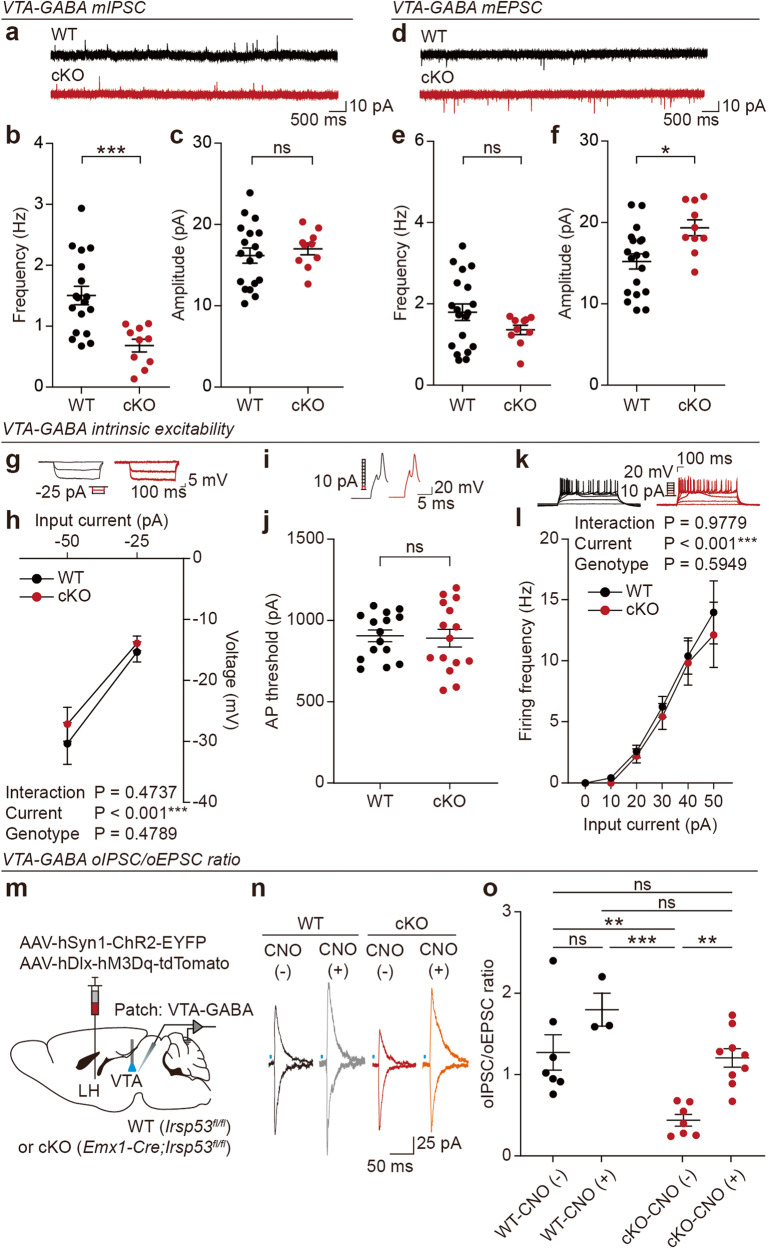
*Emx1-Cre;Irsp53*^*fl/fl*^ VTA-GABA neurons show decreased inhibition by LH-GABA neurons. **a**–**c** mIPSC frequency and amplitude in WT and *Emx1-Cre;Irsp53*^*fl/fl*^ (cKO) VTA-GABA neurons (8–12 weeks). (*n* = 18 neurons from 3 mice [WT], 10, 3 [cKO], Student’s *t* test). **d**–**f** mEPSC frequency and amplitude in WT and *Emx1-Cre;Irsp53*^*fl/fl*^ (cKO) VTA-GABA neurons (8–12 weeks). (*n* = 19, 3 [WT], 10, 3 [cKO], Student’s *t* test). **g**, **h** Normal input resistance in VTA-GABA neurons of *Emx1-Cre;Irsp53*^*fl/fl*^ mice (8–12 weeks). (*n* = 15, 3 [WT], 16, 3 [cKO], two-way ANOVA). **i**, **j** Normal AP threshold in VTA-GABA neurons of *Emx1-Cre;Irsp53*^*fl/fl*^ mice (8–12 weeks). (*n* = 15, 3 [WT], 15, 3 [cKO], Student’s *t* test). **k**, **l** Normal current-firing curve in VTA-GABA neurons of *Emx1-Cre;Irsp53*^*fl/fl*^ mice (8–12 weeks). (*n* = 12, 3 [WT], 13, 3 [cKO], two-way ANOVA). **m** Strategy to measure oIPSC/oEPSC ratios in VTA-GABA neurons receiving inputs from LH-GABA neurons by co-injection of AAV5-hSyn1-ChR2-EGFP and AAV5-hDlx-hM3Dq-tdTomato in the LH and measurement oIPSC/oEPSC ratios in VTA-GABA neurons with/without chemogenetic/CNO activation. **n** Examples of oIPSCs and oEPSCs measured in VTA-GABA neurons with/without CNO treatment for LH-GABA neuronal stimulation in WT and *Emx1-Cre;Irsp53*^*fl/fl*^ (cKO) mice (8–12 weeks). **o** oIPSC/oEPSC ratios in VTA-GABA neurons with/without CNO treatment in WT and *Emx1-Cre;Irsp53*^*fl/fl*^ (cKO) mice. (*n* = 7, 3 [WT-CNO (−)], 3, 2 [WT-CNO (+)], 7, 3 [cKO-CNO (−)], 9, 3 [cKO-CNO (+)] two-way ANOVA with Sidak’s multiple comparison test).

*Emx1-Cre;Irsp53*^*fl/fl*^ VTA-GABA neurons displayed decreased mIPSC frequency without any change in amplitude (Fig. 5a–c), suggestive of decreased inhibitory synaptic input from LH-GABA neurons. mEPSCs in these neurons had unaltered frequency but increased amplitude (Fig. 5d–f). Neuronal excitability parameters (input resistance, AP threshold, and current-firing curve) were normal in these neurons (Fig. 5g–l). These results suggest that *Emx1-Cre;Irsp53*^*fl/fl*^ VTA-GABA neurons are insufficiently inhibited.

To more directly test if the LH^GABA^-VTA^GABA^ pathway was weakened in *Emx1-Cre;Irsp53*^*fl/fl*^ mice, we measured optogenetically induced IPSCs (oIPSCs) and oEPSCs in VTA-GABA neurons receiving LH inputs. This was achieved by injecting AAV5-hSyn1-ChR2-EYFP in the LH and measuring oIPSCs/oEPSCs ratios in light-stimulated VTA neurons. Here, both GABA and glutamate LH neurons were induced to express ChR2, as this enabled us to measure oIPSC/oEPSC ratios in VTA-GABA neurons. In addition, LH neuronal ChR2 expression was combined with chemogenetic activation of LH-GABA neurons using co-injected AAV-hDlx-hM3Dq-tdTomato (hDlx for GABA neuronal gene expression [57]) to see if the weakened LH^GABA^-VTA^GABA^ pathway could be normalized (Fig. 5m). A substantial portion (~65.0 ± 8.88%) of hM3Dq-expressing LH-GABA neurons was ChR2-positive (Supplementary Fig. 10).

In the baseline condition without chemogenetic/CNO activation, mutant VTA-GABA neurons displayed a significant decrease in the oIPSC/oEPSC ratios compared with those of control (*Irsp53*^*fl/fl*^) neurons (Fig. 5n, o). Upon chemogenetic activation, mutant VTA-GABA neurons showed increased oIPSC/oEPSC ratios that were comparable to WT values.

These results collectively suggest that the LH^GABA^-VTA^GABA^ pathway is weakened in *Emx1-Cre;Irsp53*^*fl/fl*^ mice and can be normalized by chemogenetic activation of LH-GABA neurons.

### VTA-DA neurons are over-inhibited in *Emx1-Cre;Irsp53*^*fl/fl*^ mice, and optogenetic LH-GABA neuronal activation improves social interaction

We next asked whether the weakened LH^GABA^-VTA^GABA^ pathway in *Emx1-Cre;Irsp53*^*fl/fl*^ mice would over-activate VTA-GABA neurons and subsequently over-inhibit VTA-dopamine/DA neurons in the VTA^GABA^-VTA^DA^ pathway, which is known to regulate social brain functions through DA actions at the nucleus accumbens [9, 42–44, 58–60]. To test this possibility, we first measured spontaneous synaptic transmission in VTA-DA neurons of *Emx1-Cre;Irsp53*^*fl/fl*^ mice. These neurons can be identified by specific electrophysiological and morphological properties [2, 61].

**Fig. 6:**
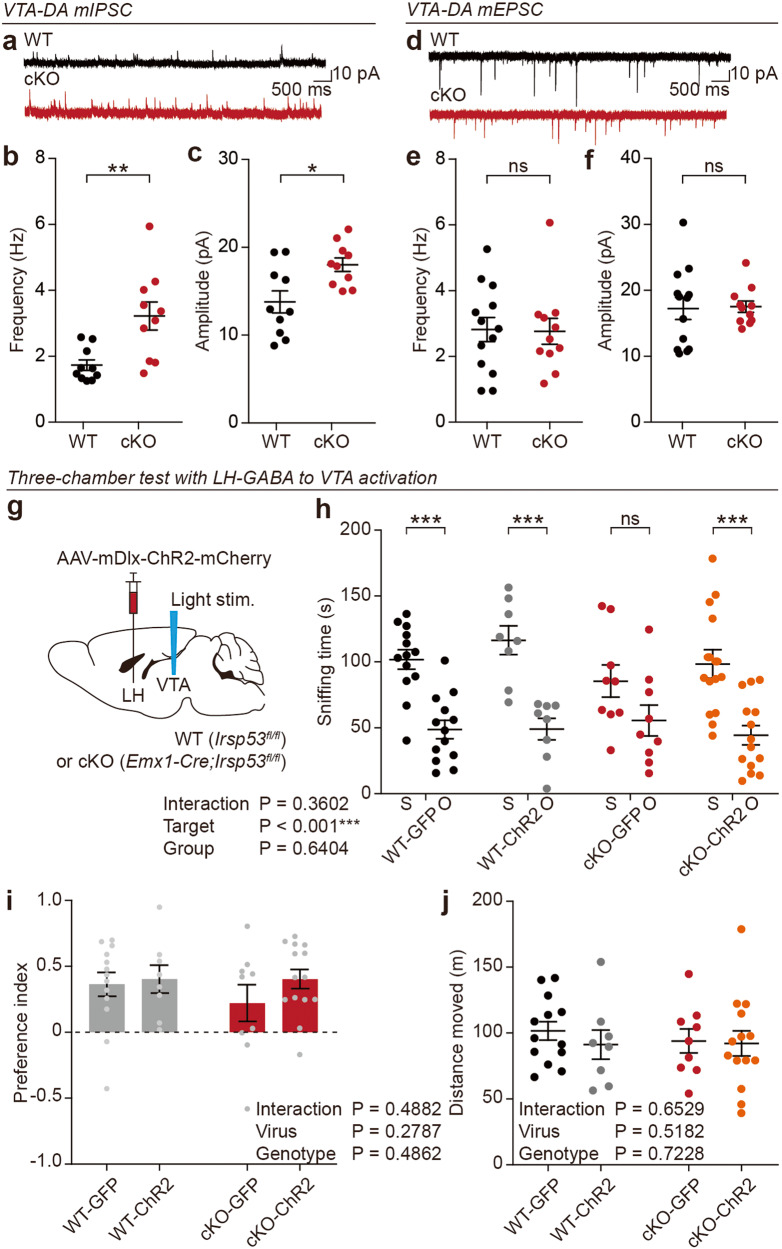
VTA-DA neurons are over-inhibited in *Emx1-Cre;Irsp53*^*fl/fl*^ mice, and optogenetic activation of LH-GABA neurons improves social interaction. **a**–**c** mIPSC frequency and amplitude in WT and *Emx1-Cre;Irsp53*^*fl/fl*^ (cKO) VTA-DA neurons (8–12 weeks). (*n* = 10 neurons from 3 mice [WT], 10, 3 [cKO], Student’s *t* test). **d**–**f** mEPSC frequency and amplitude in WT and *Emx1-Cre;Irsp53*^*fl/fl*^ (cKO) VTA-DA neurons (8–12 weeks). (*n* = 13, 3 [WT], 11, 3 [cKO], Student’s *t* test). **g** Strategy to rescue social behavior of *Emx1-Cre;Irsp53*^*fl/fl*^ mice via optogenetic activation of LH-GABA neurons; AAV-mDlx-ChR2-mCherry was injected into the LH and blue light was applied to the VTA while mice engaged in a social behavioral test (12 weeks). **h**, **i** Optogenetic activation of LH-GABA neurons improves social approach in *Emx1-Cre;Irsp53*^*fl/fl*^ mice in the three-chamber test, as shown by time spent sniffing social and object targets (S, social stranger; O, object) and the preference index (S–O over S + O). Note that there is no genotype x target interaction in the two-way ANOVA for the preference index but the S-O difference is significant on Student’s *t* test; this is indicative of a moderate rescue. (*n* = 13 [WT-GFP], 8 [WT-ChR2], 9 [cKO-GFP], 14 [cKO-ChR2], two-way ANOVA, Student’s *t* test [S-O differences in each group indicated, preference index]). **j** Optogenetic activation of LH-GABA neurons does not alter the locomotor activity of WT and cKO mice in the three-chamber test, as shown by total distance moved. (*n* = 13 [WT-GFP], 8 [WT-ChR2], 9 [cKO-GFP], 14 [cKO-ChR2] two-way ANOVA).

VTA-DA neurons in *Emx1-Cre;Irsp53*^*fl/fl*^ mice showed strong increases in the frequency and amplitude of mIPSCs (Fig. 6a–c), but no change in the frequency or amplitude of mEPSCs (Fig. 6d–f). These results suggest that VTA-DA neurons are over-inhibited in the mutant mice, potentially leading to the social deficits observed in *Emx1-Cre;Irsp53*^*fl/fl*^ mice.

If this were the case, optogenetic activation of the LH^GABA^-VTA^GABA^ pathway should improve social interaction in the mutant mice. To explore this hypothesis, we injected AAV-mDlx-ChR2-mCherry in the LH and shined blue light on the VTA in *Emx1-Cre;Irsp53*^*fl/fl*^ mice during social interaction (Fig. 6g).

Optogenetic LH-GABA neuronal activation improved the social approach of *Emx1-Cre;Irsp53*^*fl/fl*^ mice in the three-chamber test (Fig. 6h, i); this was supported by the time spent sniffing social and object targets but not by the social preference index, suggesting that there was a partial rescue. WT mice were not affected by the optogenetic LH-GABA neuronal activation. In addition, locomotor activity during the three-chamber test was not affected in WT or mutant mice (Fig. 6j).

These results collectively suggest that VTA-DA neurons are over-inhibited in *Emx1-Cre;Irsp53*^*fl/fl*^ mice, and optogenetic activation of LH-GABA neurons improves the social deficits.

## Discussion

In this study, we attempted to identify neural circuits and synaptic and neuronal changes that contribute to the social deficits observed in *Emx1-Cre;Irsp53*^*fl/fl*^ mice. Our results suggest that the mPFC-LH^GABA^-VTA^GABA^-VTA^DA^ pathway contributes to the social deficits (summarized in Supplementary Fig. 11). This hypothesis is supported by our findings that social deficits are induced by circuit-specific IRSp53 KO in the mPFC-LH^GABA^ pathway of WT mice and social rescue is induced by optogenetic activation of the LH^GABA^-VTA^GABA^-VTA^DA^ pathway in the mutant mice. Our results also indicate that cortical IRSp53 deletion leads to cortical area-, layer-, and circuit-differential changes in neuronal excitability as well as indirect changes in remote subcortical regions, including the LH and VTA, at the levels of synapses and neuronal excitability.

IRSp53 deletion restricted to cortical neurons in *Emx1-Cre;Irsp53*^*fl/fl*^ mice, which suppresses excitatory synaptic transmission (i.e., that of prelimbic layer 5 pyramidal neurons) [35, 39], leads to cortical area-, layer-, and circuit-differential changes in excitability. Specifically, increased excitability is seen in prelimbic layer 5 pyramidal neurons but not prelimbic layer 2/3 neurons. ACA and MOs layer 2/3 and 5 neurons show largely normal excitability with the exception of MOs layer 5 neurons, which show moderately decreased excitability. Moreover, LH-projecting mutant prelimbic layer 5 neurons, but not cPFC-projecting mutant prelimbic layer 5 neurons, show increased excitability. Although more research is required, these modifications appear to demonstrate (at the very least) that prelimbic layer 5 pyramidal neurons may specifically exhibit enhanced neuronal output for subcortical neurons and may therefore contribute to the social deficits observed in the mutant mice. In line with this, our recent study on in vivo single-unit recording in global IRSp53-KO mice with social deficits [33] revealed that these mice exhibited enhanced baseline firing in the mPFC (mainly prelimbic) under resting states but suppressed firing variability and cortical encoding during social interaction [36].

Another key result in the present study is that circuit-selective IRSp53 deletion in the mPFC-LH^GABA^ pathway of WT mice leads to social deficits involving the hyperexcitability of LH-projecting prelimbic layer 5 pyramidal neurons, which is similar to the changes observed in *Emx1-Cre;Irsp53*^*fl/fl*^ mice. These results suggest that there is a cell-autonomous compensatory mechanism wherein the loss of IRSp53, which is a key excitatory synaptic protein [22, 33, 62], may induce an opposite change (in this case neuronal hyperexcitability) to normalize neuronal output. Our transcriptomic analyses of the mutant mPFC identified substantial decreases in the expression of potassium channel-related genes, which would contribute to the neuronal hyperexcitability, although care should be taken because the transcriptomic results were obtained from mixed populations of mPFC neurons with different properties of projection. We previously reported that IRSp53 deletion induces a paradoxical increase in NMDAR function through abnormal actin stabilization at excitatory synapses [38, 39]. Our present results extend these findings by showing that IRSp53 deletion can induce compensatory alterations in neuronal excitability in addition to synaptic function.

Our present work offers lines of evidence suggesting that the LH^GABA^-VTA^GABA^-VTA^DA^ pathway contributes to the social deficits seen in *Emx1-Cre;Irsp53*^*fl/fl*^ mice. LH-GABA neurons display increased excitatory synaptic transmission, which appears to involve the hyperexcitability of prelimbic layer 5 neurons. This change in the mPFC-LH pathway seems to induce a secondary but opposite change in LH-GABA neurons, namely decreases in excitability and neuronal output. This is similar to the adaptive behaviors of the prelimbic layer 5 pyramidal neurons in the same mutant mice. The possible weakening of the inhibitory LH^GABA^-VTA^GABA^ pathway is supported by a decrease in the inhibitory synaptic input to VTA-GABA neurons and our observation that optogenetic activation of the LH^GABA^-VTA^GABA^ pathway normalizes synaptic inputs to VTA-GABA and VTA-DA neurons as well as social deficits.

Although our data suggest that LH-GABA neurons are an important mediator linking prelimbic layer 5 neurons with VTA-GABA/DA neurons for social deficits in *Emx1-Cre;Irsp53*^*fl/fl*^ mice, this should not be assumed to be the major pathway for the following reasons: (1) mPFC neurons project to many non-LH target regions, including the nucleus accumbens, ventral pallidum, amygdala, and VTA [5, 14, 55, 63–66]. (2) LH neurons receive inputs from many non-mPFC regions, such as the bed nucleus of the stria terminalis/BNST, lateral septum, and nucleus accumbens [67–69]. (3) VTA neurons also receive inputs from many non-LH regions, such as the mPFC, ventral pallidum, and amygdala [5, 42, 44, 60, 64, 70, 71]. Therefore, it would be fair to conclude that the mPFC-LH^GABA^-VTA^GABA^-VTA^DA^ pathway may partially contribute to the social deficits observed in *Emx1-Cre;Irsp53*^*fl/fl*^ mice. This probably explains why a circuit-specific IRSp53 deletion in the mPFC-LH pathway does not fully impair social interaction in WT mice, and why optogenetic activation of LH-GABA neurons does not fully rescue the social deficits in the mutant mice. Whether the mPFC-LH-VTA pathway regulates social function in WT mice remains an open question, although this possibility is supported by our data derived from circuit-specific IRSp53 KO in WT mice. More generally and perhaps more importantly, our results suggest that changes occurring in cortical output neurons could significantly impact various subcortical social-related areas in mouse models of ASD. This could be applicable to brain diseases other than ASD, given that IRSp53/BAIAP2 has been implicated in schizophrenia [29, 30] and ADHD [31, 32], in addition to ASD [26–28].

In conclusion, our data collectively suggest that IRSp53 KO restricted to cortical glutamatergic neurons in mice leads to social deficits and widespread indirect changes in synaptic and neuronal properties of different brain regions, including the LH and VTA, and that modulation of the mPFC-LH-VTA pathway contributes to and can be harnessed to improve social deficits in IRSp53-mutant mice.

### Reporting summary

Further information on research design is available in the Nature Research Reporting Summary linked to this article.

### Supplementary information

Supplementary Information

Source Data 1

Supplementary Table 1

Supplementary Table 2

Supplementary Table 3

Editorial policy checklist

Reporting summary

Reproducibility checklist

## Data Availability

The source data underlying the graphs in the main and supplementary figures are provided as a Source Data file (Source Data 1).
