# Supplementary material for: The PFC-LH-VTA pathway contributes to social deficits in IRSp53-mutant mice
Source: Mol Psychiatry. 2023 Sep 20;28(11):4642–54. doi: 10.1038/s41380-023-02257-y (PMC10914623; doi:10.1038/s41380-023-02257-y)
Supplement: Supplementary file 1 — Supplementary Information [file 41380_2023_2257_MOESM1_ESM.docx]

**SUPPLEMENTARY INFORMATION**

**The PFC-LH-VTA pathway contributes to social deficits in IRSp53-mutant mice**

Young Woo Noh^1,*^, Yangsik Kim^2,*^, Soowon Lee^3^, Yeonghyeon Kim^1^, Jae Jin Shin^4^, Hyojin Kang^5^, Il-Hwan Kim^6^, Eunjoon Kim^1,4,#^

^1^Department of Biological Sciences, Korea Advanced Institute of Science and Technology (KAIST), Daejeon 34141, Korea;

^2^Department of Psychiatry, Inha University Hospital, Incheon 22332, Korea; ^3^Graduate School of Medical Science and Engineering, KAIST, Daejeon 34141, Korea; ^4^Center for Synaptic Brain Dysfunctions, Institute for Basic Science, Daejeon 34141, Korea; ^5^Division of National Supercomputing, Korea Institute of Science and Technology Information (KISTI), Daejeon 34141, Korea; ^6^Department of Anatomy and Neurobiology, University of Tennessee Health Science Center, Memphis, TN 38163, USA

^#^Correspondence: kime@kaist.ac.kr

**Supplementary materials and methods**

**Animals**

Mice were fed ad libitum, and 2–6 mice were housed together in a cage under a 12-hour light/dark cycle. There were no differences in the body weights of age-matched mouse groups. Mice were identified by PCR genotyping using the following PCR primers: IRSp53 flox AGGAGGTGTTTCTGCTCTGG / AATAGCAGTCTGGGGTCTGG; Cre CGTACTGACGGTGGGAGAAT/ TGCATGATCTCCGGTATTGA.

**Behavioral assays**

All behavioral assays were performed using age-matched C57BL6/J mice (8-16 weeks) generated by Cre/+;*Irsp53^fl/fl^* x *Irsp53^fl/fl^* mating. All behavioral assays were performed during light-off periods. Light condition for all behavioral assays was ~120 lux. There were at least one day-long rest periods between tests. The behavioral assays were performed in the order of the open-field test, elevated plus-maze test, light-dark test, and three-chamber social interaction test. Behavioral assays were recorded as video files (.avi format) and analyzed by EthoVision XT 13 (Noldus, Netherlands)

**Three-chamber social interaction test**

The three-chamber social interaction test was performed as described previously ^1, 2^. The apparatus had the following dimensions; W 60 x H 40 x D 20 cm for the whole apparatus, and W 20 x H 20 x D 20 cm for each chamber. The side chambers contained an aluminium grid with a curved face to confine mouse/object.

The assay consisted of three sessions. During the first 10-minute session (Empty-Empty, E-E), a subject mouse was allowed to freely explore all three chambers for habituation. Then the mouse was confined briefly in the center chamber, while a novel object and a WT stranger mouse (stranger 1) were placed in the side chambers behind the aluminium grid (Stranger 1-Object, S1-O). The subject mouse was then allowed to freely explore all three chambers for 10 min. Before the last session, the subject mouse was again gently guided to the center chamber while the object was replaced with another WT mouse (stranger 2). The subject mouse was again allowed to freely explore all three chambers for 10 min (S1-Stranger2, S1-S2). All stranger mice were age-matched 129/SvJae males and were habituated to the side chambers in advance during the previous day for 30 min. The positions of the object and stranger mouse were alternated between tests to minimize the influences of side preference.

For optogenetic modulation of LH-GABA to VTA, we used 473 nm laser, with a 4-4.5 mW power at the tip to illuminate VTA region. We applied 20 Hz of light stimulation to activate ChR2 on the VTA. Light stimulation was delivered to the mouse brain during the mice in the three-chamber (10 min for E-E session, and 10 min for S1-O session). We applied a minute of light off period between sessions. S1-S2 session was omitted for the purpose of reducing brain damage caused by light stimulation.

**Open-field test**

Mice were placed in the center region of an open-field box (W 40 x H 40 x D 40 cm). Open-field locomotor activities were measured for 60 min. The center zone was defined as a square of 20 x 20 cm in the center of the box. The activity of mice including distance moved and time stayed in the center zone was measured using EthoVision XT13

**Elevated plus-maze test**

An elevated plus-maze was made of grey acryl with four elevated arms, each 30 cm long and 5 cm wide ^3^. The elevated height of the maze was 75 cm above the ground. Closed arms were enclosed by 30 cm-height walls, and the light condition of closed arms was ~0 lux. A test mouse was placed in the center of the maze at the junction of the four arms in the beginning and was allowed to explore the maze for 10 min.

**Light-dark test**

The light area consists of a white acryl chamber (W 20 x H 30 x D 20 cm) without a ceiling, and the light area is connected to an enclosed, black acryl chamber (W 20 x H 13 x D 20 cm) with an entrance that allows mice to freely move between the two chambers. The light illumination intensity of the light chamber was ~300 lux. Time spent in the light area was recorded for 10 min. Entry into the light chamber was counted only when the mouse’s entire body crossed the entrance.

**Whole-cell recordings**

Coronal slices (mPFC, NAc, and LH) and horizontal slices (VTA) were prepared using a vibratome (VT1200S, Leica, Germany) in ice-cold dissection buffer (SCSF; in mM: 212 sucrose, 25 NaHCO_3_, 5 KCl, 1.25 NaH_2_PO_4_, 10 D-glucose, 2 sodium pyruvate, 1.2 sodium ascorbate, 3.5 MgCl_2_, 0.5 CaCl_2_ bubbled with 95% O2/ 5% CO_2_). The slices were recovered at 32 ºC in normal artificial cerebrospinal fluid (ACSF; in mM: 125 NaCl, 25 NaHCO_3_, 2.5 KCl, 1.25 NaH_2_PO_4_, 10 D-glucose, 1.3 MgCl_2_, 2.5 CaCl_2_) and thereafter maintain at room temperature. Cells were visualized using infrared differential interference contrast video microscopy (Olympus, BX50XI). Whole-cell current-clamp recordings were made by using a MultiClamp 700B amplifier (Molecular Devices).

For voltage-clamp recordings, recording pipettes (3–5 MΩ) were filled with a solution containing (in mM) 120 CsMeSO_4_, 15 CsCl, 10 TEA-Cl, 8 NaCl, 10 HEPES, 0.25 EGTA, 5 QX-314, 4 MgATP, and 0.3 NaGTP, pH 7.25–7.35 (280–300 mOsm) ^4^. Signals were filtered at 2 kHz and digitized at 10 kHz. mEPSCs were recorded in the presence of AP5 (50 μM) and tetrodotoxin (1 μM) at the holding potential of -70 mV. mIPSCs were recorded at the holding potential of 0 mV, as described previously ^5^. sEPSCs and sIPSCs were recorded in the presence of AP5 (50 μM).

Optogenetically evoked EPSCs and IPSCs (oEPSCs and oIPSCs) were recorded at the holding potential of -70 mV and 0 mV, respectively. After obtaining a stable baseline of oEPSCs for 10 min, the holding potential was shifted to 0 mV to record oIPSCs. Ten consecutive traces were averaged to obtain mean currents of oEPSCs and oIPSCs. To stimulate ChR2-expressing LH-GABA axon fibers, light from 470 nm light emitting diode (LED) (T-cube, Thorlab, NJ) was emitted from objective lens mounted LED of the electrophysiology set-up. Triggered pulses of blue light (5-ms duration; 6 mW/mm^2^ under the objective) were delivered at the recording site with 30-sec intervals ^6^.

For current-clamp recordings, recording pipettes (3–5 MΩ) were filled with a solution containing (in mM) 120 Kgluconate, 20 HEPES, 0.4 EGTA, 2.8 NaCl, 5 TEA-Cl, 2.5 MgATP, and 0.25 NaGTP, pH 7.25–7.35 (280–300 mOsm). Picrotoxin (100 μM) and NBQX (10 μM) were present throughout the experiments to block inhibitory and excitatory synaptic transmissions, respectively. If the series resistance changed by more than 20%, data were not included in the analysis. Membrane potentials were not corrected for junction potentials (estimated to be 10 mV). To obtain sustained firings, a series of current (1 s duration, 50 pA steps for mPFC and NAc, 10 pA steps for LH and VTA-GABA) was injected. To measure action potential thresholds, a series of current steps (2 ms duration at 2.5 Hz, 0 to 2500 pA range, +10 pA step increments) were injected into patched neurons until an action potential was generated. To measure the input resistance, hyperpolarizing current steps (1 s duration, 0 to −100 pA, −25 pA step increments) were injected into patched neurons. All voltage measures were taken after neurons had reached a stable response ^7^.

GABAergic neurons in the LH were selected using the following criteria; cells with small soma and cell capacitance < 40 pF, which showed larger input resistance and lower AP threshold in our current-clamp recordings, unlike glutamatergic neurons, as previously published ^8^. GABAergic neurons in the VTA were selected using the following criteria; cells with small soma and cell capacitance < 40 pF, which, upon hyperpolarization steps (−60 mV, 500 ms duration immediately after whole-cell patch clamp configuration) in our voltage-clamp recordings, did not induce *I*_h_ currents, unlike dopaminergic neurons, as previously reported ^9^. DA neurons in the VTA were selected using the following criteria; cells with large soma and cell capacitance > 40 pF, which, upon hyperpolarization steps (−60 mV, 500 ms duration immediately after whole-cell patch clamp configuration) in our voltage-clamp recordings, induce *I*_h_ currents, as previously reported ^9, 10^. For LH-GABA oEPSC and mPFC projection specific patch clamp recordings, we additionally used fluoresence expressed by AAV to identify specific cell types.

**Stereotaxic brain surgery for viral vectors and**

For stereotaxic brain surgery, mice were anesthetized with 4% volume/volume isoflurane and placed in a stereotaxic apparatus (Kopf instruments, CA, US). During the stereotaxic surgery, 1-1.5 % v/v isoflurane was continuously delivered with oxygen gas. Stereotaxic injections were performed as described previously ^11^. Head fur was shaved and small incision was made. Craniotomy was made above the mouse brain region of interest using a dental drill, using the following coordinates; mPFC (Anterior-posterior (AP) +1.8 mm, Medio-lateral (ML) ±0.3 mm, and Dorso-ventral (DV) -1.5 mm, from Bregma) LH (AP −1.3 mm, ML ± 1.0 mm, DV −5.0 mm) ^12^, VTA (AP -3.3 mm, ML ± 0.5mm, DV -4.4 mm). AAV were infused at a rate of 0.1 μl per min, and delayed 8-10 min for the diffusion after infusion. After the surgery, mice were placed in the recovery chamber for > 30 min. Behavioral tests were performed at least 5 weeks after stereotaxic virus injections. Optogenetically evoked synaptic transmissions were measured ~8 weeks after stereotaxic virus injections.

The viral vectors used in the present study are as follows: For retrograde tracing and mPFC projection type specific electrophysiology, AAVrg-hSyn-EGFP and AAVrg-hSyn-mCherry (Addgene) were used. For circuit selective KO-related experiments, AAV(PHP.eB)-EF1a-CreN-InteinN, AAVrg-EF1a-InteinC-CreC (Institute for basic science (IBS) virus facility), AAV5-hSyn-DIO-mCherry, and AAVrg-EF1a-EGFP (Addgene) were used. For LH-GABA oEPSC, AAV5-CamKIIa-ChR2 (H134R)-EYFP (Addgene) and AAV (PHP.eB)-mDlx-EGFP (IBS) were used. For oIPSC/oEPSC ratio measurement, AAV5-hSyn-hM3D(Gq)-mCherry, AAV5-hSyn-mCherry (Control) and AAV5-hSyn-hChR2 (H134R)-EYFP were used. Viral vectors were aliquoted and stored at −80°C.

**RNA-Seq analysis**

Five mice aged P56 were used for each group (conditional knock-out, wildtype). Brains were quickly dissected and deep-freezed in RNAlater solution (Ambion) to stabilize RNAs. RNA extraction, library preparation, cluster generation, and sequencing were conducted by Theragen Bio Inc. Sequencing was performed with an average read depth of 40 to 60 million reads at paired-ends (2 × 151 bp) using an Illumina Novaseq 6000 (Illumina) via Theragen Bio Inc. Transcript abundance was estimated in pseudo-mapping-based mode for the Mus musculus genome (GRCm38) using Salmon (v1.1.0) ^13^. Differential gene expression analysis was performed using R/Bioconductor DEseq2 (v1.30.1) ^14^ by importing the estimated abundance data into R (v.4.1.3) using the tximport package ^15^. The p-values were adjusted for multiple testing with the Benjamini-Hochberg correction. Genes with an adjusted P value of less than 0.05 were considered as differentially expressed.

**Gene set enrichment analysis**

Gene Set Enrichment Analysis (GSEA) (http://software.broadinstitute.org/gsea) ^16^ was used to capture if the expressions of genes in a specific gene set are changed in a consistent direction, although each might not be significant enough to be counted as differentially expressed genes. Enrichment analysis was performed using GSEAPreranked (gsea-4.2.3.jar) module on gene set collections downloaded from Molecular Signature Database (MSigDB) v7.5.1 (broadinstitute.org). GSEAPreranked was applied using the list of all genes expressed, ranked by the fold change and multiplied by the inverse of the P value with recommended default settings (1,000 permutations and a classic scoring scheme). The gene sets with an FDR of less than 0.05 were considered as significantly enriched. Integration and visualization of the GSEA results were performed using the EnrichmentMap Cytoscape App (version 3.9.1) ^17, 18^.

**Quantitative PCR**

Total RNAs were extracted from the mPFC, somatomotor area, and Lateral hypothalamus region of each mouse using Trizol solution (Invitrogen). 1 µg of total RNA was used for cDNA synthesis using M-MLV cDNA synthesis kit (Enzynomics, EZ006M). mRNA expression levels were quantified using SYBR-green PCR mix (Toyobo, QPK-201) and a real-time PCR machine (Bio-rad, CFX96). The primer sequences are as follows: GAPDH: 5’-CATCACTGCCACCCAGAAGACTG-3’ and 5’-ATGCCAGTGAGCTTCCCGTTCAG-3’; IRSp53: 5’-TCTACAAGACCATCATGGAGCA-3’ and 5’-CCCCCATCTTTACCAGAGCA-3’; KCNK5: 5’-CTATTCCTTCATCACCATCTC-3’ and 5’-AGCCCCAGGTAGATCCAA-3’; KCNK3:

5’-TCATCACCACAATCGGCTAT-3’ and 5’AGCGCGTAGAACATGCAGAA-3’; KCNQ2: 5’-AGTCCAAGAGCAGCATCGGCAA-3’ and 5’-CAGTGACTGTCCGCTCGTAGTA-3’;

KCNQ3: 5’-AAGCCTACGCTTTCTGGCAGAG-3’ and 5’-ACAGCTCGGATGGCAGCCTTTA-3’.

**Data availability**

Raw RNA-Seq results are available as GSE221836 at GEO (Gene Expression Omnibus), NCBI (National Center for Biotechnology Information). The source data underlying the graphs in Figs 1–6 and Supplementary Figs 1, 2, 5, and 7–9 are provided as a Source Data file (**Source Data 1**).

**References for supplementary methods**

1. Silverman JL, Yang M, Lord C, Crawley JN. Behavioural phenotyping assays for mouse models of autism. *Nat Rev Neurosci* 2010; **11**(7)**:** 490-502.

2. Moy SS, Nadler JJ, Perez A, Barbaro RP, Johns JM, Magnuson TR *et al.* Sociability and preference for social novelty in five inbred strains: an approach to assess autistic-like behavior in mice. *Genes Brain Behav* 2004; **3**(5)**:** 287-302.

3. Walf AA, Frye CA. The use of the elevated plus maze as an assay of anxiety-related behavior in rodents. *Nat Protoc* 2007; **2**(2)**:** 322-328.

4. Rothwell PE, Fuccillo MV, Maxeiner S, Hayton SJ, Gokce O, Lim BK *et al.* Autism-associated neuroligin-3 mutations commonly impair striatal circuits to boost repetitive behaviors. *Cell* 2014; **158**(1)**:** 198-212.

5. Liang J, Xu W, Hsu YT, Yee AX, Chen L, Sudhof TC. Conditional neuroligin-2 knockout in adult medial prefrontal cortex links chronic changes in synaptic inhibition to cognitive impairments. *Mol Psychiatry* 2015; **20**(7)**:** 850-859.

6. Peixoto RT, Wang W, Croney DM, Kozorovitskiy Y, Sabatini BL. Early hyperactivity and precocious maturation of corticostriatal circuits in Shank3B(-/-) mice. *Nat Neurosci* 2016; **19**(5)**:** 716-724.

7. Chen BT, Yau HJ, Hatch C, Kusumoto-Yoshida I, Cho SL, Hopf FW *et al.* Rescuing cocaine-induced prefrontal cortex hypoactivity prevents compulsive cocaine seeking. *Nature* 2013; **496**(7445)**:** 359-362.

8. Karnani MM, Szabo G, Erdelyi F, Burdakov D. Lateral hypothalamic GAD65 neurons are spontaneously firing and distinct from orexin- and melanin-concentrating hormone neurons. *J Physiol* 2013; **591**(4)**:** 933-953.

9. Bariselli S, Tzanoulinou S, Glangetas C, Prevost-Solie C, Pucci L, Viguie J *et al.* SHANK3 controls maturation of social reward circuits in the VTA. *Nat Neurosci* 2016; **19**(7)**:** 926-934.

10. Neuhoff H, Neu A, Liss B, Roeper J. I(h) channels contribute to the different functional properties of identified dopaminergic subpopulations in the midbrain. *J Neurosci* 2002; **22**(4)**:** 1290-1302.

11. Cetin A, Komai S, Eliava M, Seeburg PH, Osten P. Stereotaxic gene delivery in the rodent brain. *Nat Protoc* 2006; **1**(6)**:** 3166-3173.

12. Sweeney P, Yang Y. An Inhibitory Septum to Lateral Hypothalamus Circuit That Suppresses Feeding. *J Neurosci* 2016; **36**(44)**:** 11185-11195.

13. Patro R, Duggal G, Love MI, Irizarry RA, Kingsford C. Salmon provides fast and bias-aware quantification of transcript expression. *Nat Methods* 2017; **14**(4)**:** 417-419.

14. Love MI, Huber W, Anders S. Moderated estimation of fold change and dispersion for RNA-seq data with DESeq2. *Genome Biol* 2014; **15**(12)**:** 550.

15. Soneson C, Love MI, Robinson MD. Differential analyses for RNA-seq: transcript-level estimates improve gene-level inferences. *F1000Res* 2015; **4:** 1521.

16. Subramanian A, Tamayo P, Mootha VK, Mukherjee S, Ebert BL, Gillette MA *et al.* Gene set enrichment analysis: a knowledge-based approach for interpreting genome-wide expression profiles. *Proc Natl Acad Sci U S A* 2005; **102**(43)**:** 15545-15550.

17. Merico D, Isserlin R, Stueker O, Emili A, Bader GD. Enrichment map: a network-based method for gene-set enrichment visualization and interpretation. *PLoS One* 2010; **5**(11)**:** e13984.

18. Isserlin R, Merico D, Voisin V, Bader GD. Enrichment Map - a Cytoscape app to visualize and explore OMICs pathway enrichment results. *F1000Res* 2014; **3:** 141.

**SUPPLEMENTARY FIGURES AND FIGURE LEGENDS**

**Supplementary Figure 1.** **Neuronal excitability in layer 2/3 and 5 neurons in the prelimbic, ACA, and MOs areas of the mPFC.**

(a–c) Prelimbic layer 2/3 neurons in *Emx1-Cre;Irsp53^fl/fl^* (cKO) mice (8–12 weeks) show normal excitability, as supported by analysis of the current-firing curve (a), action potential (AP) threshold (b), and input resistance (c). (n = 14 neurons from 3 mice [WT], 13, 3 [cKO], two-way ANOVA [current-firing curve and input resistance], Student’s t-test [AP threshold]).

(d–f) ACA layer 2/3 pyramidal neurons in *Emx1-Cre;Irsp53^fl/fl^* mice (8–12 weeks) show normal excitability, as supported by analysis of the current-firing curve, AP threshold, and input resistance. (Input resistance: n = 11, 3 [WT], 10, 3 [cKO], two-way ANOVA; AP threshold: n = 11, 3 [WT]. 11, 3 [cKO], Student’s t-test; Sustained firing: n = 11, 3 [WT], 11, 3 [cKO], two-way ANOVA).

(g–i) ACA layer 5 pyramidal neurons in *Emx1-Cre;Irsp53^fl/fl^* mice (8–12 weeks) show normal excitability. (Input resistance: n = 12, 3 [WT], 10, 3 [cKO], two-way ANOVA, AP threshold: n = 12, 3 [WT], 10, 3 [cKO], Student’s t-test; Sustained firing: n = 12, 3 [WT], 10, 3 [cKO], two-way ANOVA).

(j–l) MOs layer 2/3 pyramidal neurons in *Emx1-Cre;Irsp53^fl/fl^* mice (8–12 weeks) show normal excitability. (Input resistance: n = 14, 4 [WT], 14, 3 [cKO], two-way ANOVA, AP threshold: n = 15, 4 [WT]. 15, 3 [cKO], Student’s t-test; Sustained firing: n = 15, 4 [WT], 15, 3 [cKO], two-way ANOVA).

(m–o) MOs layer 5 pyramidal neurons in *Emx1-Cre;Irsp53^fl/fl^* mice (8–12 weeks) show decreased excitability. (Input resistance: n = 15, 4 [WT], 17, 3 [cKO], two-way ANOVA, AP threshold: n = 15, 4 [WT]. 17, 3 [cKO], Mann-Whitney test; Sustained firing: n = 15, 4 [WT], 17, 3 [cKO], two-way ANOVA).

**
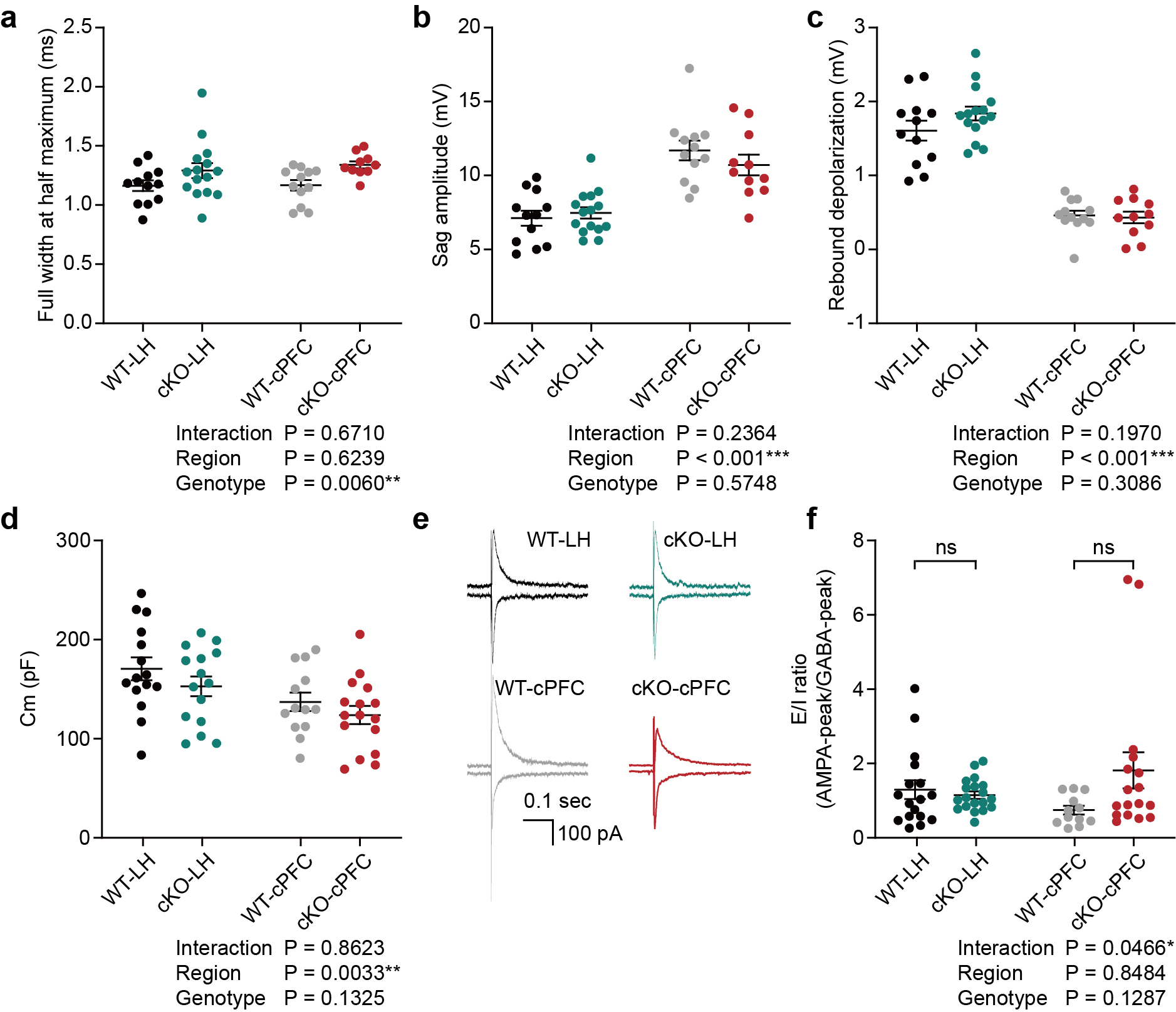
**

**Supplementary Figure 2. LH- and cPFC-projecting prelimbic neurons of *Emx1-Cre;Irsp53^fl/fl^* mice show normal properties in other measures of excitability.**

(a–d) Normal AP-related parameters (full width a half maximum, sag amplitude, rebound depolarization, and capacitance) in LH- and cPFC-projecting prelimbic layer 5 pyramidal neurons in *Emx1-Cre;Irsp53^fl/fl^* (cKO) mice labeled by AAVrg-hSyn1-EGFP and AAVrg-hSyn1-mCherry injected into LH and cPFC (8–12 weeks), respectively. (n = 12 neurons from 3 mice [WT-LH], 15, 3 [cKO-LH], 12, 3 [WT-cPFC], 10, 3 [cKO-cPFC], two-way ANOVA for FWHM, n = 12, 3 mice [WT-LH], 15, 3 [cKO-LH], 12, 3 [WT-cPFC], 11, 3 [cKO-cPFC], two-way ANOVA for sag amplitude and rebound depolarization, n = 15, 3 mice [WT-LH], 15, 3 [cKO-LH], 13, 3 [WT-cPFC], 16, 3 [cKO-cPFC], two-way ANOVA for Cm).

(e and f) Normal ratios of evoked AMPA receptor- and GABA receptor-mediated currents in LH- and cPFC-projecting prelimbic layer 5 pyramidal neurons of *Emx1-Cre;Irsp53^fl/fl^* mice, as labeled by injection of AAVrg-hSyn1-EGFP and AAVrg-hSyn1-mCherry into the LH and cPFC (8 –12 weeks), respectively. (n = 17, 4 [WT-LH], 19, 3 [cKO-LH], 12, 4 [WT-cPFC] 17, 3 [cKO-cPFC], two-way ANOVA with Sidak’s multiple comparison test).

**
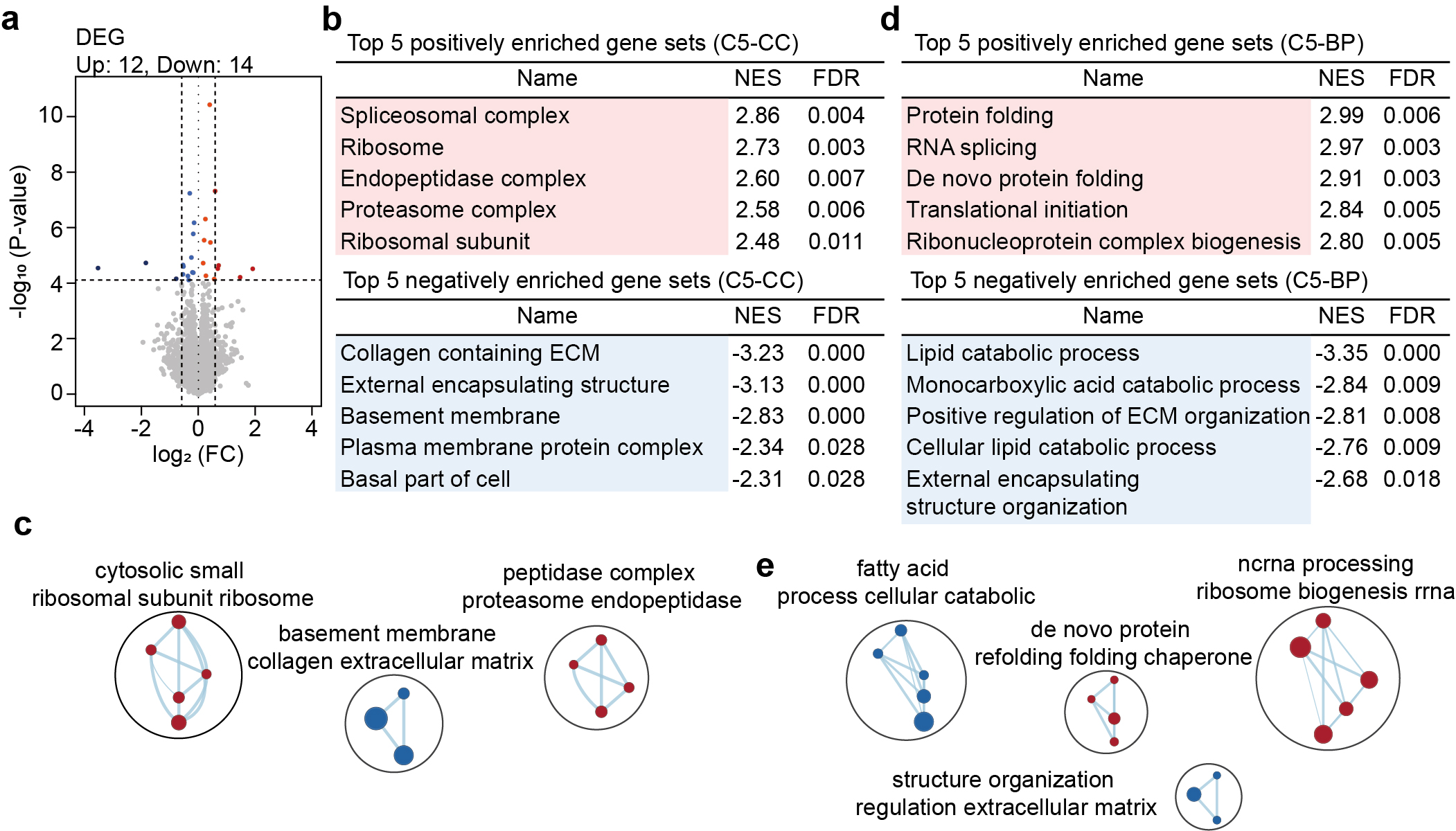
**

**Supplementary Figure 3. Volcano plot from DEG analysis and top-five enriched gene sets and their clusters from the GSEA of cKO/WT transcripts.**

(a) A volcano plot of differentially expressed genes (DEGs; Adjusted p-value < 0.05, |FC| > 1.5) derived from RNA-Seq analysis of WT and *Emx1-Cre;Irsp53^fl/fl^* (cKO) mPFC (12 weeks). (n = 5 mice [WT and cKO]; FDR < 0.05).

(b) Lists of top-five positively and negatively enriched gene sets derived from the GSEA of cKO/WT transcripts for cellular components (CC) using the GSEA-C5 ontology gene sets.

(c) Clustering of positively and negatively enriched gene sets derived from the GSEA of cKO/WT transcripts for CC, as performed using Cytoscape App EnrichmentMap.

(d) Lists of top-five positively and negatively enriched gene sets derived from the GSEA of cKO/WT transcripts for biological processes (BP) using the GSEA-C5 ontology gene sets.

(e) Clustering of positively and negatively enriched gene sets derived from the GSEA of cKO/WT transcripts for BP, as performed using Cytoscape App EnrichmentMap.

**
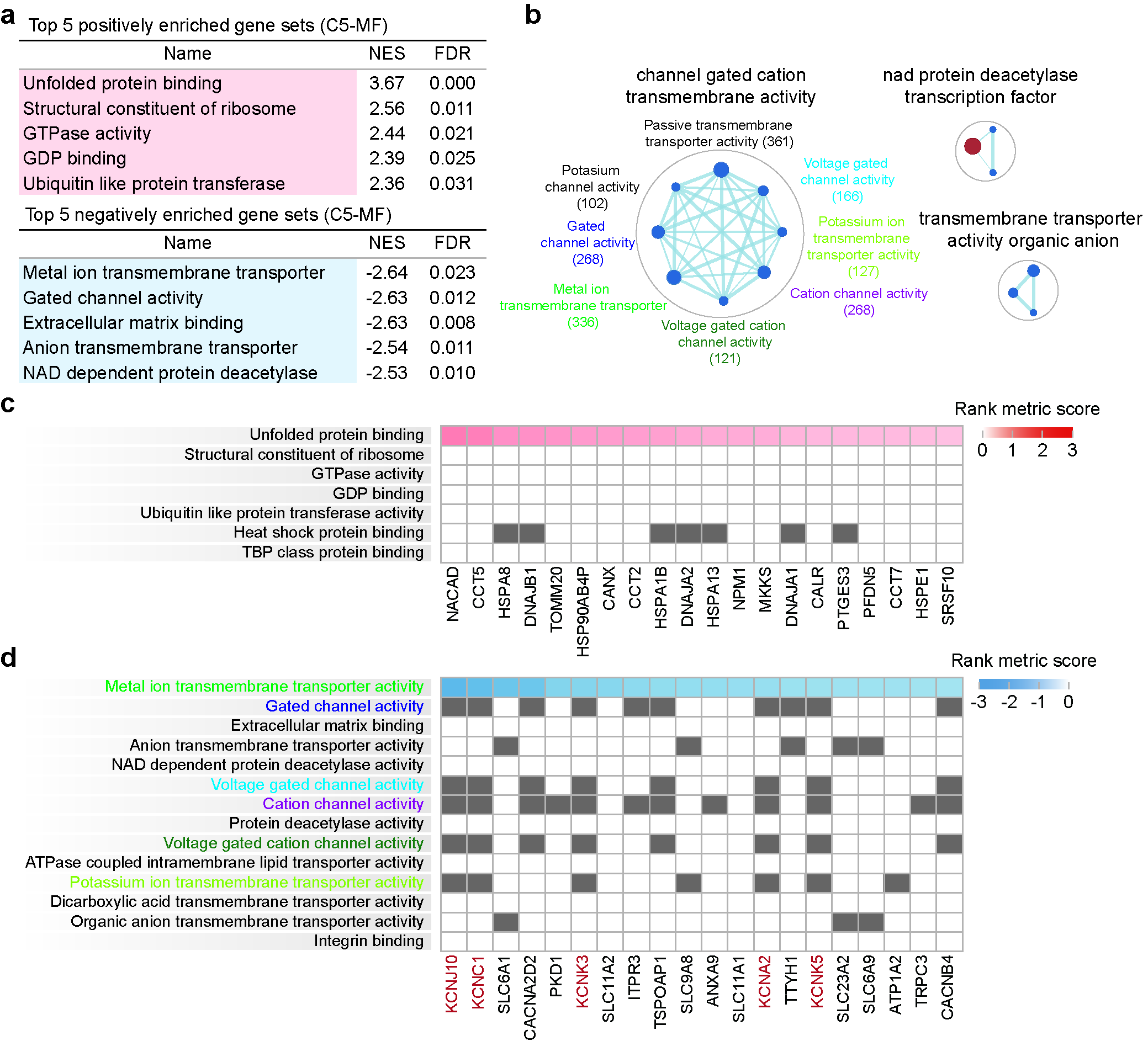
**

**Supplementary Figure 4.** **Transcriptomic changes involving potassium channel genes in the *Emx1-Cre;Irsp53^fl/fl^* mPFC.**

(a) Lists of top-five positively and negatively enriched gene sets derived from the GSEA of cKO/WT mPFC transcripts for molecular functions (MF) using the GSEA-C5 ontology gene sets. NES, normalized enrichment score; FDR, false detection rate.

(b) Clustering of positively and negatively enriched gene sets derived from the GSEA of cKO/WT transcripts for MF, using Cytoscape App EnrichmentMap (a p-value of 0.005 and FDR/false discovery rate q-value of 0.1 were used as cutoffs to select gene sets to be used in the clustering; an overlap coefficient of 0.5 was used as a cutoff for similarity between gene sets used for leading-edge analysis). The size of each gene set in the figure represents the number of genes in that set. Red and blue colors denote positive and negative enrichment, respectively. Gene sets in the ‘channel gated cation transmembrane activity’ cluster are color-coded to match those in panel c. (n = 5 mice [WT and cKO]).

(c and d) Leading-edge analysis using all negatively and positively enriched gene-set clusters derived from the GSEA of cKO/WT transcripts for the molecular function (MF) domain of the GSEA-C5 ontology gene sets. The gene list at the bottom of the matrix denotes those that strongly contribute to the enrichment to the first gene set (metal ion transmembrane transporter), a gene set with the strongest NES in (b). Note that potassium channel genes (indicated in red) are frequently identified in the leading-edge analysis of negatively enriched gene sets, but not positively enriched gene sets. (n = 5 mice [WT and cKO]).

**
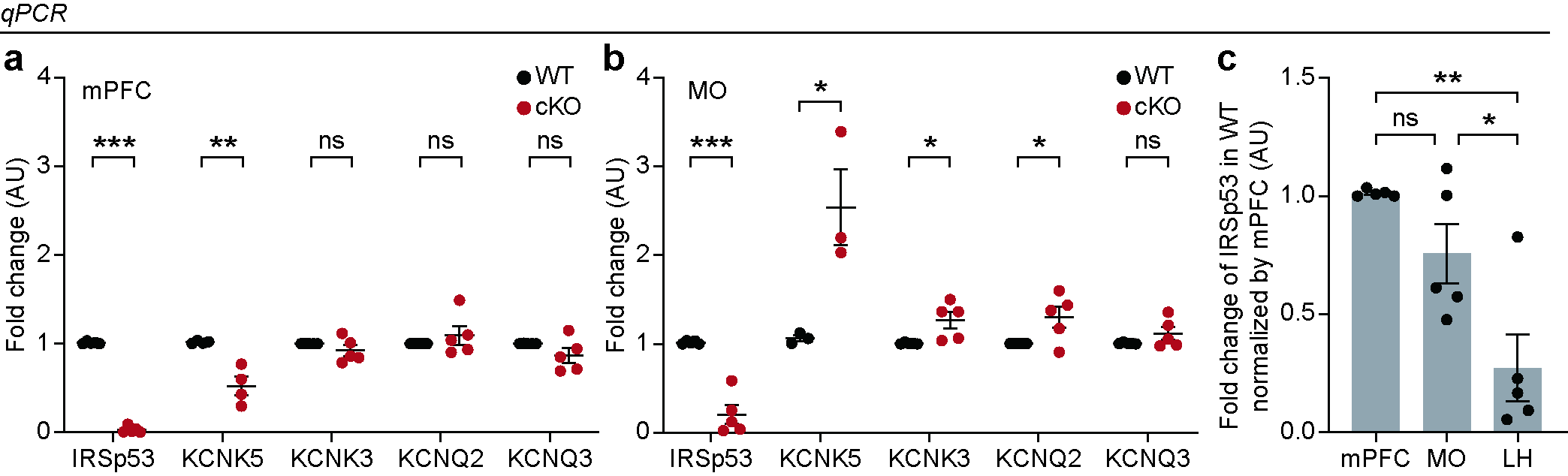
**

**Supplementary Figure 5. mRNA expression of IRSp53 and potassium channels**

(a) mRNA level of IRSp53 and KCNK5 were decreased in mPFC of *Emx1-Cre;Irsp53^fl/fl^* (cKO) mice. Note that the basemean of KCNK5 was low (14.24, 2874.87 for IRSp53, Supplementary Table 1) in RNA sequencing (n = 5 [WT-IRSp53], 5 [cKO-IRSp53], 4 [WT-KCNK5], 4 [cKO-KCNK5], 5 [WT-KCNK3], 5 [cKO-KCNK3], 5 [WT-KCNQ2], 5 [cKO-KCNQ2], 5 [WT-KCNQ3], 5 [cKO-KCNQ3], student’s t-test [IRSp53, KCNK5, KCNK3, KCNQ2], Mann-Whitney test [KCNQ3]).

(b) mRNA level of IRSp53 was decreased in Somatomotor area (MO) of cKO mice, whereas KCNK5, KCNK3, and KCNQ2 level were increased in cKO mice (n = 5 [WT-IRSp53], 5 [cKO-IRSp53], 3 [WT-KCNK5], 3 [cKO-KCNK5], 5 [WT-KCNK3], 5 [cKO-KCNK3], 5 [WT-KCNQ2], 5 [cKO-KCNQ2], 5 [WT-KCNQ3], 5 [cKO-KCNQ3], student’s t-test [IRSp53, KCNK5, KCNK3, KCNQ2, KCNQ3]).

(c) IRSp53 mRNA expression level in mPFC, MO, and Lateral hypothalamus (LH) of WT mice. Fold changes in MO and LH were normalized by expression level in mPFC (n = 5 [PFC], 5 [MO], 5 [LH], one-way ANOVA with Sidak’s multiple comparison test).

**
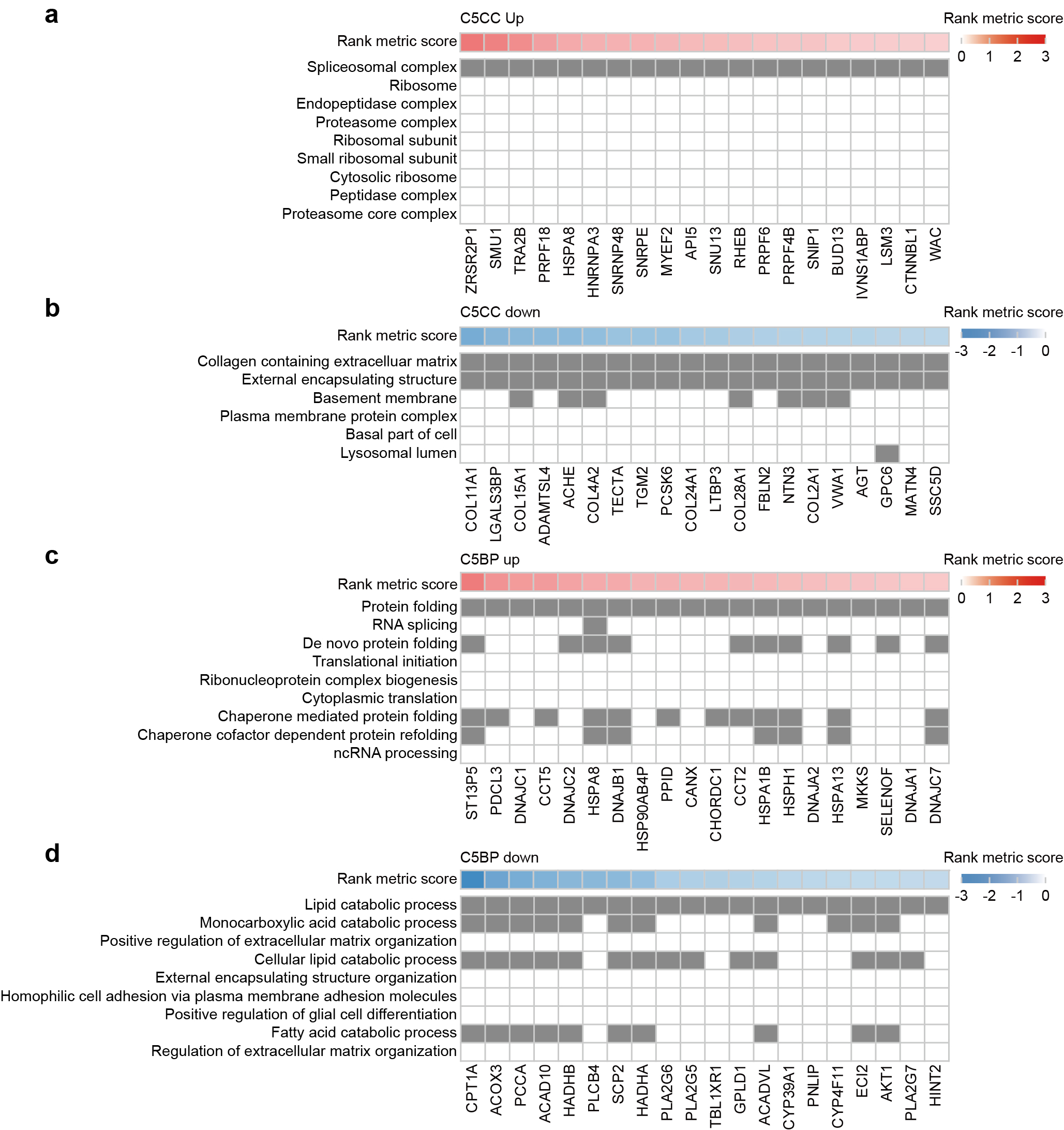
**

**Supplementary Figure 6. Leading-edge genes in the enriched gene-set clusters derived from the GSEA of cKO/WT transcripts for cellular components and biological processes.**

(a and b) Leading-edge analysis using the gene-set clusters derived from the GSEA of cKO/WT transcripts for the cellular component (CC) domain of the GSEA-C5 ontology gene sets. The genes listed at the bottom of the matrix denote those that strongly contribute to the enrichment to the gene set with a strong NES. (n = 5 mice [WT and cKO]).

(c and d) Leading-edge analysis of the gene-set clusters derived from GSEA of the cKO/WT transcripts for biological processes (BP) using the GSEA-C5 ontology gene sets. (n = 5 mice [WT and cKO]).

**
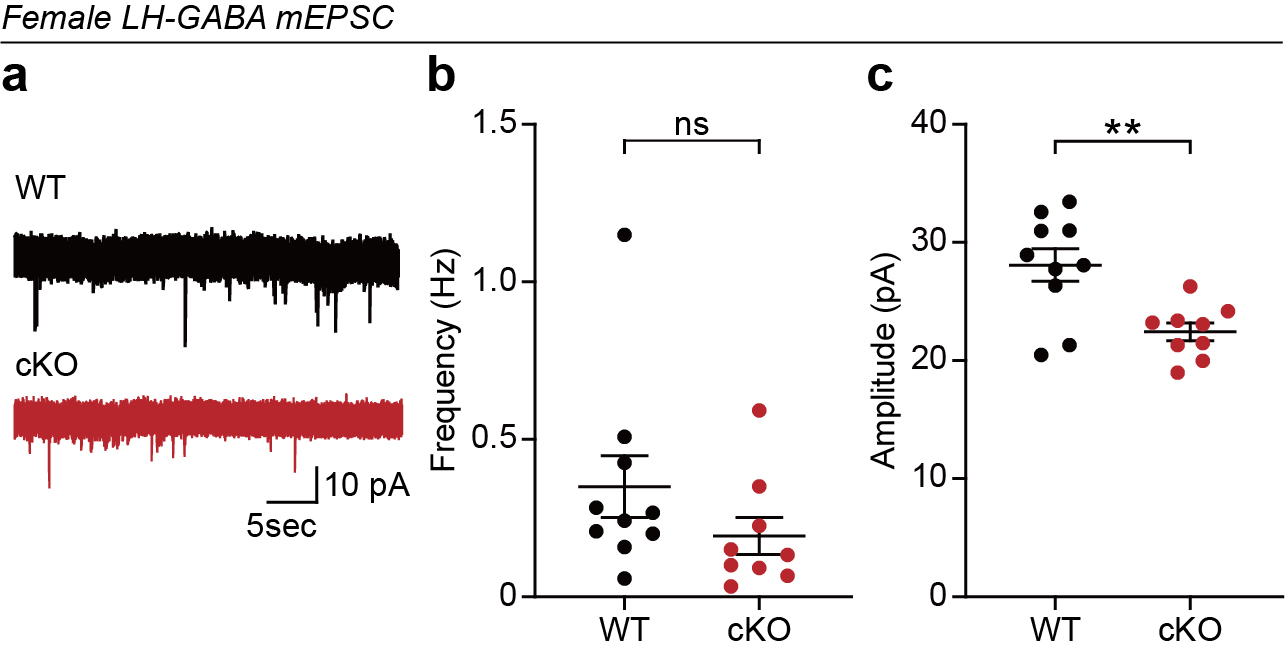
**

**Supplementary Figure 7. Female *Emx1-Cre;Irsp53^fl/fl^* mice show decreased mEPSC amplitude in LH-GABA neurons.**

(a–c) Decreased amplitude but normal frequency of mEPSCs in LH-GABA neurons of female *Emx1-Cre;Irsp53^fl/fl^* mice (3 months). (n = 10 neurons from 3 mice [f/f], 9, 3 [cKO], Mann-Whitney test [frequency], Student's t-test [amplitude]).


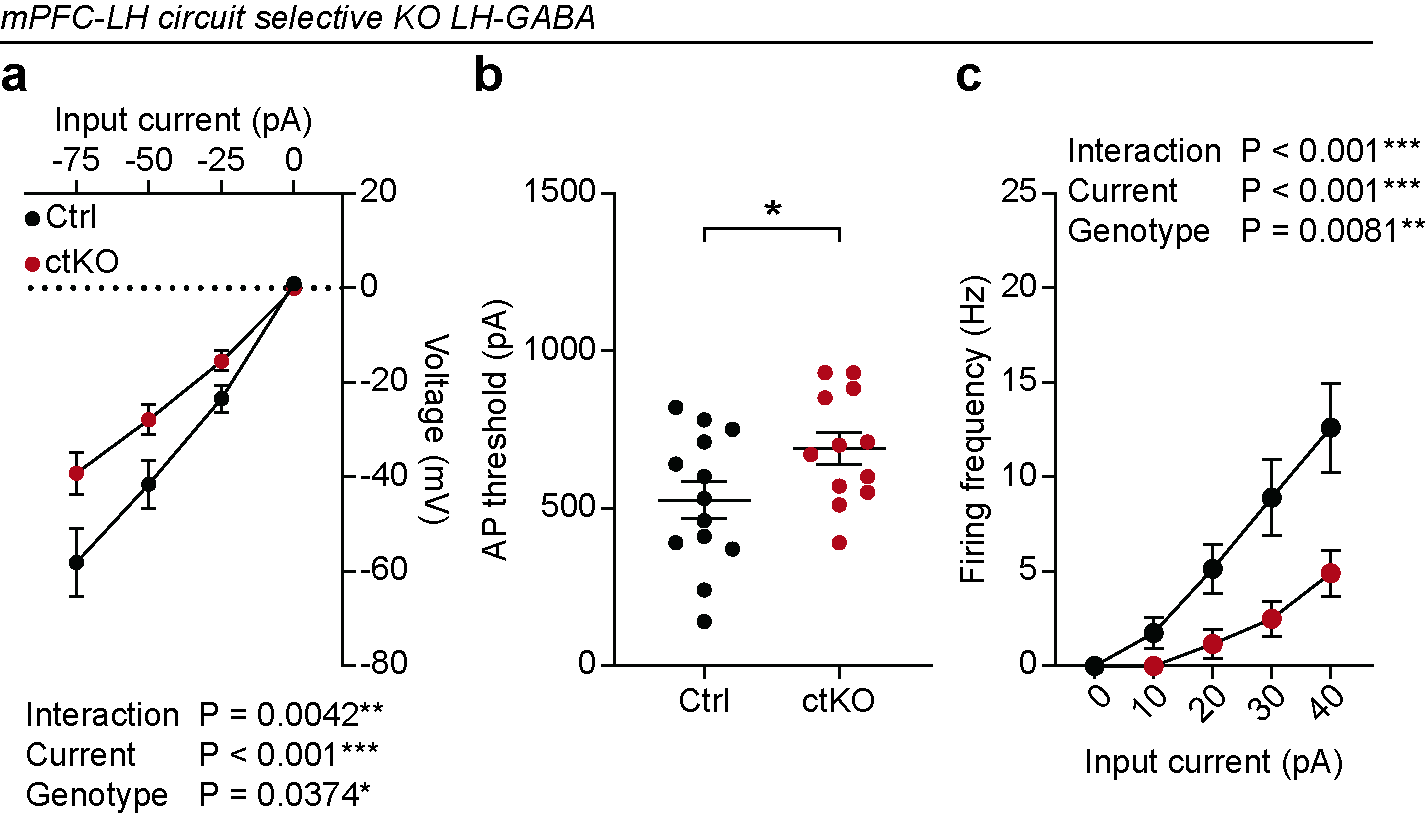


**Supplementary Figure 8. Circuit-selective deletion of IRSp53 in the mPFC leads to decreased excitability in LH-GABA neurons.**

(a) Decreased input resistance of LH-GABA neurons in mPFC-LH circuit-selective KO (ctKO). (n = 13 neurons, 3 mice [WT], 12, 3 [ctKO], two-way ANOVA with Sidak’s multiple comparison test).

(b) Increased action potential threshold in ctKO LH-GABA neurons (n = 13, 3 [WT], 12, 3 [ctKO], Student’s t-test).

(c) Decreased current-firing curve in ctKO LH-GABA neurons. Firing frequency was increased in ctKO mice (n = 13, 3 [WT], 12, 3 [ctKO], two-way ANOVA with Sidak’s multiple comparison test).

**
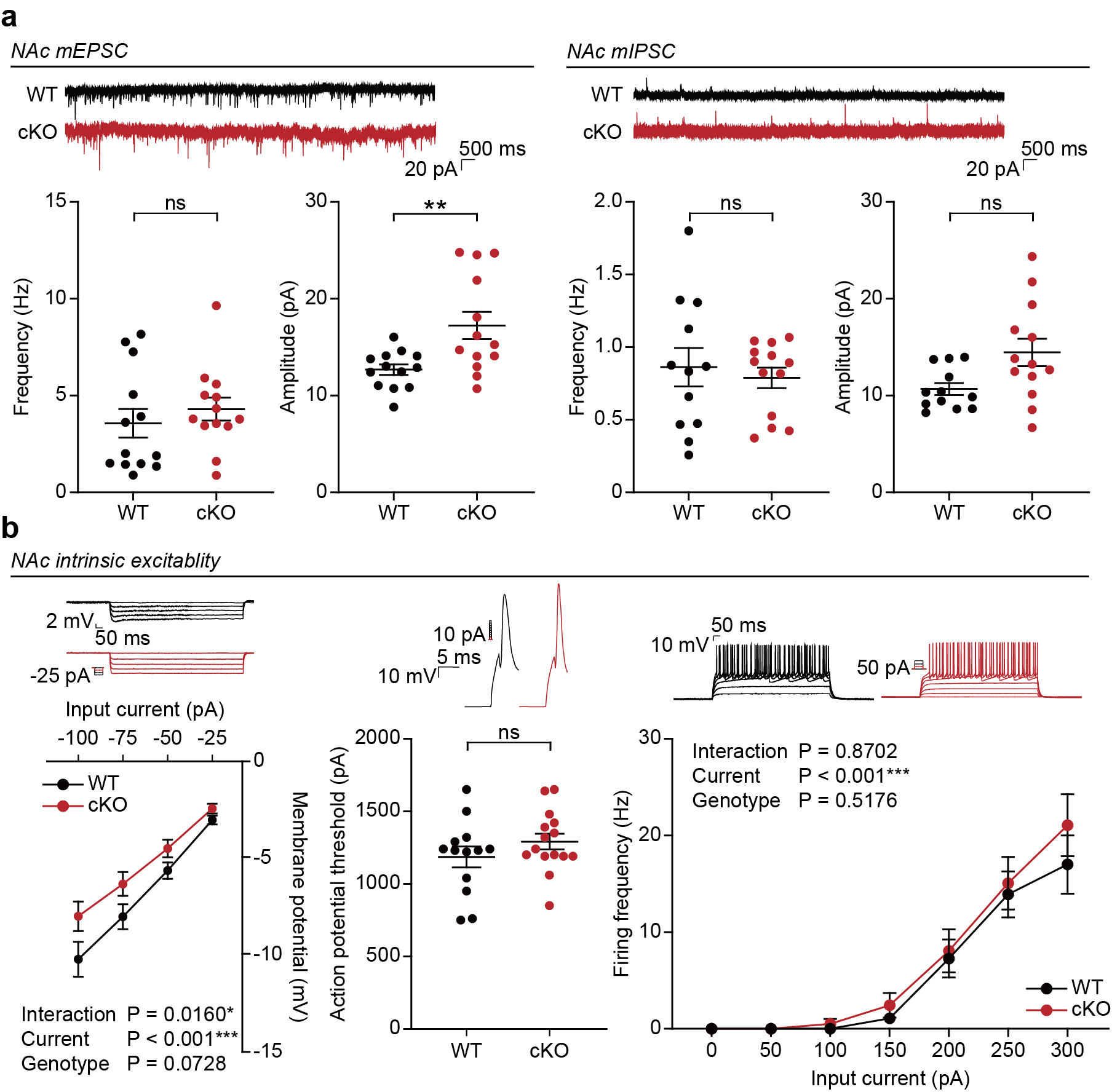
**

**Supplementary Figure 9. *Emx1-Cre;Irsp53^fl/fl^* neurons in the nucleus accumbens show altered synaptic transmission and neuronal excitability.**

(a) Increased mEPSC amplitude but normal mEPSC frequency and mIPSC frequency and amplitude are found for nucleus accumbens neurons in the ventral striatum of *Emx1-Cre;Irsp53^fl/fl^* and control (*Irsp53^fl/fl^*) mice (3 months). (n = 13 neurons from 3 mice [f/f], 13, 3 [cKO] for mEPSC, n = 12, 3 [f/f], 13, 3 [cKO] for mIPSC, Student’s t-test).

(b) Normal excitability in nucleus accumbens neurons of *Emx1-Cre;Irsp53^fl/fl^* and control (*Irsp53^fl/fl^*) mice (3 months), as shown by input resistance, AP threshold, and current-firing curve. (n = 13, 3 [f/f-input resistance], 16, 3 [cKO-input resistance], 13,3 [f/f-AP threshold], 15, 3 [cKO-AP threshold], 11, 3 [f/f-current-firing curve] 16, 3 [cKO-current-firing curve], Student’s t-test and two-way ANOVA with Bonferroni's test).

Significance is indicated as * (< 0.05), ** (< 0.01), *** (< 0.001), or ns (not significant).

**
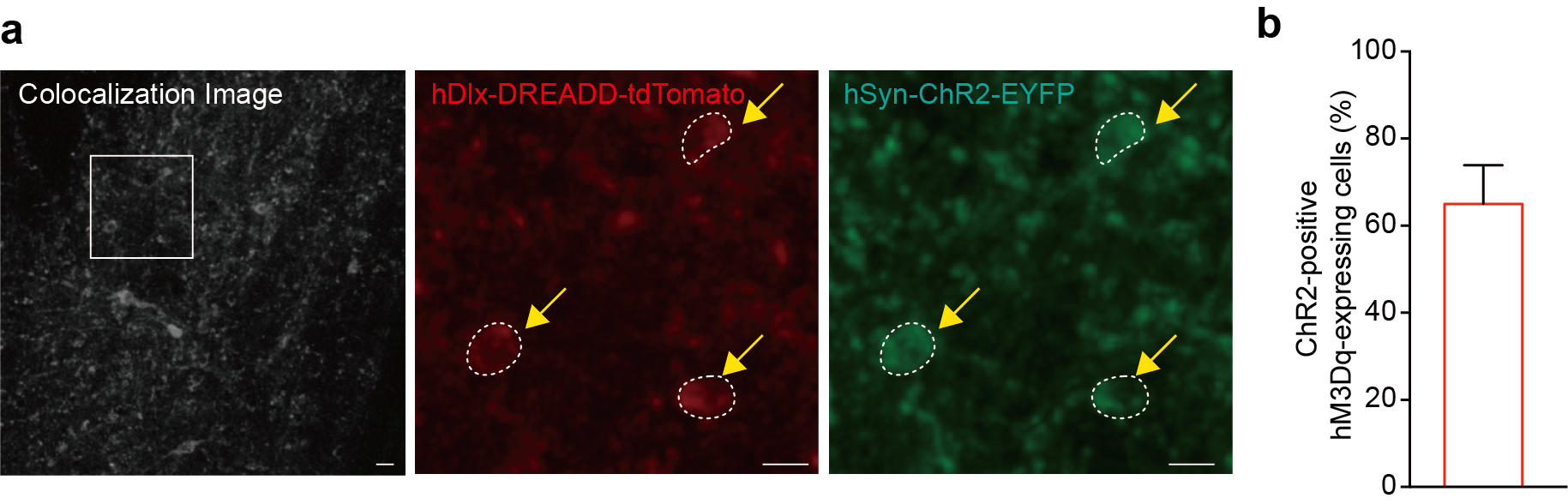
**

**Supplementary Figure 10. Colocalization between hM3Dq- and ChR2-expressing LH neurons.**

(a) An example of the colocalization between hM3Dq- and ChR2-expressing neurons in the LH. The LH region in control (*Irsp53^fl/fl^*) mice was injected with AAV5-hDlx-hM3Dq-mCherry and AAV-hSyn-ChR2-EGFP, and double fluorescence staining was performed for mCherry and EYFP. Scale bar, 20 μm

(b) Quantification of the results in (a), as shown by the percentage of EGFP (ChR2)-positive cells in mCherry (hM3Dq)-positive cells. (n = 3 mice).

**
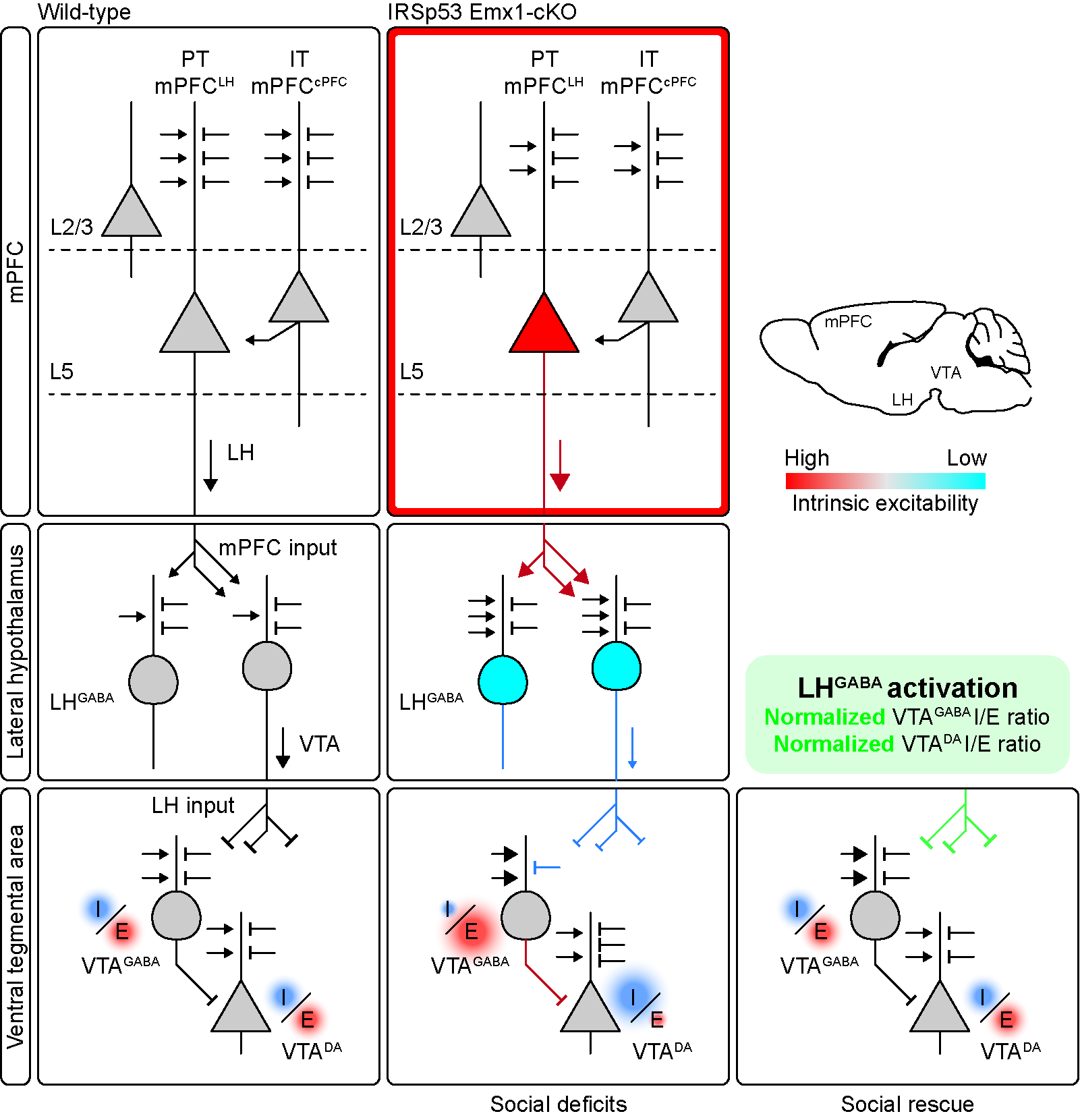
**

**Supplementary Figure 11.** **Summary of current findings and working hypotheses.** IRSp53 deletion in the mPFC of *Emx1-Cre;Irsp53^fl/fl^* mice leads to an abnormal increase in the excitability of LH-projecting mPFC layer 5 neurons (shown in red) in a circuit-specific manner. This leads to excessive excitatory synaptic inputs onto target LH-GABA neurons, which seems to lead a compensatory decrease in the excitability of LH-GABA neurons (shown in blue). This weakens the inhibitory LH^GABA^-VTA^GABA^ pathway, leading to abnormal activation of VTA-GABA neurons and abnormal inhibition of VTA-DA neurons, as shown by altered synaptic I/E (inhibition/excitation) ratios. These changes would collectively lead to social deficits in the mutant mice via an insufficient DA release in the nucleus accumbens. These changes can be rescued by the optogenetic activation of LH-GABA neurons and subsequent normalizations in the I/E ratios in VTA-GABA and VTA-DA neurons. mPFC, medial prefrontal cortex; L2/3 and L5, layers 2/3 and 5; LH, lateral hypothalamus, VTA, ventral tegmental area, DA, dopamine, I/E, synaptic inhibition-excitation. **Supplementary tables**

**Supplementary Table 1. Raw RNA-Seq data from the WT and *Emx1-Cre;Irsp53^fl/fl^* (cKO) mPFC.**

**Supplementary Table 2. List of DEGs for the cKO/WT transcripts.**

**Supplementary Table 3. Enriched gene sets from the GSEA of cKO/WT transcripts.**
