# Supplementary material for: The PFC-LH-VTA pathway contributes to social deficits in IRSp53-mutant mice
Source: Mol Psychiatry. 2023 Sep 20;28(11):4642–54. doi: 10.1038/s41380-023-02257-y (PMC10914623; doi:10.1038/s41380-023-02257-y)
Supplement: Supplementary file 7 — Reporting summary [file 41380_2023_2257_MOESM7_ESM.pdf]

nature portfolio

Corresponding author(s): Eunjoon Kim

Last updated by author(s): Aug 22, 2023

Reporting Summary

Nature Portfolio wishes to improve the reproducibility of the work that we publish. This form provides structure for consistency and transparency in reporting. For further information on Nature Portfolio policies, see our Editorial Policies and the Editorial Policy Checklist.

Statistics

For all statistical analyses, confirm that the following items are present in the figure legend, table legend, main text, or Methods section.

n/a	Confirmed	
☐	☒	The exact sample size (*n*) for each experimental group/condition, given as a discrete number and unit of measurement
☐	☒	A statement on whether measurements were taken from distinct samples or whether the same sample was measured repeatedly
☐	☒	The statistical test(s) used AND whether they are one- or two-sided<br>*Only common tests should be described solely by name; describe more complex techniques in the Methods section.*
☒	☐	A description of all covariates tested
☐	☒	A description of any assumptions or corrections, such as tests of normality and adjustment for multiple comparisons
☐	☒	A full description of the statistical parameters including central tendency (e.g. means) or other basic estimates (e.g. regression coefficient) AND variation (e.g. standard deviation) or associated estimates of uncertainty (e.g. confidence intervals)
☐	☒	For null hypothesis testing, the test statistic (e.g. *F*, *t*, *r*) with confidence intervals, effect sizes, degrees of freedom and *P* value noted<br>*Give P values as exact values whenever suitable.*
☒	☐	For Bayesian analysis, information on the choice of priors and Markov chain Monte Carlo settings
☒	☐	For hierarchical and complex designs, identification of the appropriate level for tests and full reporting of outcomes
☒	☐	Estimates of effect sizes (e.g. Cohen's *d*, Pearson's *r*), indicating how they were calculated

Our web collection on statistics for biologists contains articles on many of the points above.

Software and code

Policy information about availability of computer code

Data collection	Clampex 10.6 and pCLAMP 9 were used to acquire in vitro electrophysiological data
Data analysis	All behavioral data were analyzed using EthoVision XT13 (Noldus) and Imaging data were analyzed using ImageJ and Amasine software. All statistics were analyzed using Prism 9.1.2 (Graphpad)

For manuscripts utilizing custom algorithms or software that are central to the research but not yet described in published literature, software must be made available to editors and reviewers. We strongly encourage code deposition in a community repository (e.g. GitHub). See the Nature Portfolio guidelines for submitting code & software for further information.

Data

Policy information about availability of data

All manuscripts must include a data availability statement. This statement should provide the following information, where applicable:

- Accession codes, unique identifiers, or web links for publicly available datasets
- A description of any restrictions on data availability
- For clinical datasets or third party data, please ensure that the statement adheres to our policy

The data that support the findings of this study are available from the corresponding author upon reasonable request.

Human research participants

Policy information about studies involving human research participants and Sex and Gender in Research.

Reporting on sex and gender	No human data were included in this study.
Population characteristics	No human data were included in this study.
Recruitment	No human data were included in this study.
Ethics oversight	No human data were included in this study.

Note that full information on the approval of the study protocol must also be provided in the manuscript.

Field-specific reporting

Please select the one below that is the best fit for your research. If you are not sure, read the appropriate sections before making your selection.

☒ Life sciences ☐ Behavioural & social sciences ☐ Ecological, evolutionary & environmental sciences

For a reference copy of the document with all sections, see nature.com/documents/nr-reporting-summary-flat.pdf

Life sciences study design

All studies must disclose on these points even when the disclosure is negative.

Sample size	Sample sizes were chosen based on our previous studies using the same IRSp53 mutant (IRSp53 fl/fl; Emx1-Cre) mice (Kim et al., Frontiers in cellular neuroscience 23, 2020, and Noh et al., Communications Biology, 2022), and global IRSp53 mutant mice (Chung et al., Nature neuroscience 435-443, 2015).
Data exclusions	Outliers were excluded using the Outlier test (ROUT, with Q=1% of aggressiveness).
Replication	All experiments were replicated through multiple cohort/mice analysis.
Randomization	We counterbalanced side (left-right) factor for all behavior test, where applicable.
Blinding	All experiments and analysis were performed with blind manner for all mouse genotype or treated virus type.

Reporting for specific materials, systems and methods

We require information from authors about some types of materials, experimental systems and methods used in many studies. Here, indicate whether each material, system or method listed is relevant to your study. If you are not sure if a list item applies to your research, read the appropriate section before selecting a response.

Materials & experimental systems

n/a	Involved in the study	
☒	☐	Antibodies
☒	☐	Eukaryotic cell lines
☒	☐	Palaeontology and archaeology
☐	☒	Animals and other organisms
☒	☐	Clinical data
☒	☐	Dual use research of concern

Methods

n/a	Involved in the study	
☒	☐	ChIP-seq
☒	☐	Flow cytometry
☒	☐	MRI-based neuroimaging

Animals and other research organisms

Policy information about studies involving animals; ARRIVE guidelines recommended for reporting animal research, and Sex and Gender in Research

Laboratory animals	C57BL/6J mice were used for all experiments in this study, except social target in three chamber test (age and sex matched 129/SvJae mice), Adult (8-14 weeks) male mice were used. All animals were fed ad libitum, and 4-6 mice were housed together with out

	genotype separation.
Wild animals	No wild animals were used.
Reporting on sex	Only male mice were used for all behavioral experiments, and parts of electrophysiology experiments, except for female LH-GABAergic neuronal property measurement.
Field-collected samples	No Field-collected samples were used.
Ethics oversight	The animal study was reviewed and approved by the Committee of Animal Research at KAIST (KA2022-059).

Note that full information on the approval of the study protocol must also be provided in the manuscript.
